# CENPA functions as a transcriptional regulator to promote hepatocellular carcinoma progression via cooperating with YY1

**DOI:** 10.7150/ijbs.85656

**Published:** 2023-10-16

**Authors:** Jingyu Liao, Zeyu Chen, Ruizhi Chang, Tong Yuan, Ganxun Li, Chang Zhu, Jingyuan Wen, Yi Wei, Zhao Huang, Zeyang Ding, Liang Chu, Junnan Liang, Bixiang Zhang

**Affiliations:** 1 Hepatic Surgery Center, Tongji Hospital, Tongji Medical College, Huazhong University of Science and Technology, Wuhan, China.; 2 Clinical Medical Research Center of Hepatic Surgery at Hubei Province, Wuhan, China.; 3 Hubei Key Laboratory of Hepato-Pancreatic-Biliary Diseases, Tongji Hospital, Tongji Medical College, Huazhong University of Science and Technology, Wuhan, China.

**Keywords:** CENPA, YY1, Lactylation, HCC

## Abstract

The centromere proteins (CENPs), a critical mitosis-related protein complexes, are involved in the kinetochore assembly and chromosome segregation. In this study, we identified that CENPA was significantly up-regulated in HCC and highly expressed CENPA correlated with poor prognosis for HCC patients. Knockdown of CENPA inhibited HCC cell proliferation and tumor growth *in vitro* and *in vivo*. Mechanistically, CENPA transcriptionally activated and cooperated with YY1 to drive the expression of cyclin D1 (CCND1) and neuropilin 2 (NRP2). Moreover, we identified that CENPA can be lactylated at lysine 124 (K124). The lactylation of CENPA at K124 promotes CENPA activation, leading to enhanced expression of its target genes. In summary, CENPA function as a transcriptional regulator to promote HCC via cooperating with YY1. Targeting the CENPA-YY1-CCND1/NRP2 axis may provide candidate therapeutic targets for HCC.

## Introduction

Hepatocellular carcinoma (HCC) is one of the most common cancer diagnoses and the main causes of cancer-related deaths worldwide 1. It is a fatal malignant tumor with significant histological and biological heterogeneity. The current therapeutic options mainly include surgery, chemotherapy, immunotherapy, radiation and interventional therapy 2, 3. Although various diagnostic methods and multiple therapies for HCC patients have proven effective, the 5-year overall survival (OS) rate is still less than 50%. Thus, more investigations are critical to fully understanding the molecular mechanism underlying HCC pathogenesis.

CENPs proteins are a family of highly conserved mitosis-related proteins, including CENPA, CENPB, CENPC, etc., which play essential roles in kinetochore formation, chromosome segregation, cell division, cell cycle regulation, and tumorigenesis 4-7. Recent research revealed that the expression level of CENPs is higher in multiple types of cancers 8-10, which indicated that CENPs are potential markers associated with the development of tumors 11, 12.

Among the family members of CENPs, CENPA is a variant of histone H3 and plays a critical role in providing the foundation for the assembly of the outer kinetochore 5, 13-15. Previously, CENPA was considered to be linked to centromere maintenance and correct chromosome segregation by associating with various components of the Constitutive Centromere-Associated Network (CCAN) 16, 17. For example, loading new CENPA to the previous CENPA deposition site is restricted to mitosis and G1 phase, and long-term stability of the centromeric mark is achieved by effective recycling of expelled CENPA by Spt6 18. Recently, accumulating evidence indicates that CENPA is also involved in the transcription activation process and is closely related to tumorigenesis 19. Bioinformatic analysis identified that CENPA was a transcription regulator in prostate cancer 20. However, the function of CENPA in HCC tumorigenesis remains unclear, and the underlying mechanism of its role needs further research.

Protein post-transcriptional modification (PTM) influence fundamental properties and cellular biological process, including gene transcription, protein attachment, metabolic reprogramming and so on 21, 22. Several types of PTM with essential functions have been identified on CENPA, including phosphorylation, ubiquitylation, methylation, and acetylation 23-26. For example, the phosphorylation of serine 68 generates a potential steric hindrance and impairs the interaction between CENPA and HJURP, while this interaction is facilitated by lysine124 ubiquitylation 27. Recently, lactate-derived lactylation of histone lysine residues has been identified as a new PTM directly involved in multiple biological processes and drives tumor progression 28, 29. Zhang et al. identified histone lactylation can activate gene transcription 29. The lactylation of PKM2 at K62 was also reported to drive the macrophages transited to a reparative phenotype 30. However, whether there exists this kind of new modification on CENPA needs further research.

In this study, we demonstrated that CENPA was up-regulated in HCC, and the high expression of CENPA predicts poor prognosis of HCC patients. We identified CENPA function as a transcription factor, and the lactylation modification of CENPA enhanced its transcriptional activation ability. Moreover, CENPA interacted with YY1 to function as a co-transcriptional complex to regulate a set of oncogenes, including CCND1 and NRP2.

## Methods

### Patients and tissue specimen

We obtained 100 pairs of HCC tissues and adjacent liver tissues from patients with HCC who underwent surgery between 2015 and 2016 at Hepatic Surgery Centre, Tongji Hospital of Huazhong University of Science and Technology (Wuhan, China). (Tongji cohort). We obtained another 37 paired frozen samples from the Tongji cohort to establish the tissue microarray (TMA).

OS time was defined as the period between surgical resection and death or last follow-up. Recurrence free survival (RFS) was determined from the date of hepatectomy to tumor recurrence or last follow-up. The study protocol confirmed to the ethical guidelines of the 1975 Declaration of Helsinki and was approved by the Ethics Committee of Tongji Hospital, Tongji Medical College, Huazhong University of Science and Technology (Wuhan, China) (TJ-IRB20210924).

Detailed materials and methods are listed in the Supplementary information.

## Results

### CENPA is highly expressed in human HCC tissues and correlated with poor prognosis in HCC

**Figure 1:**
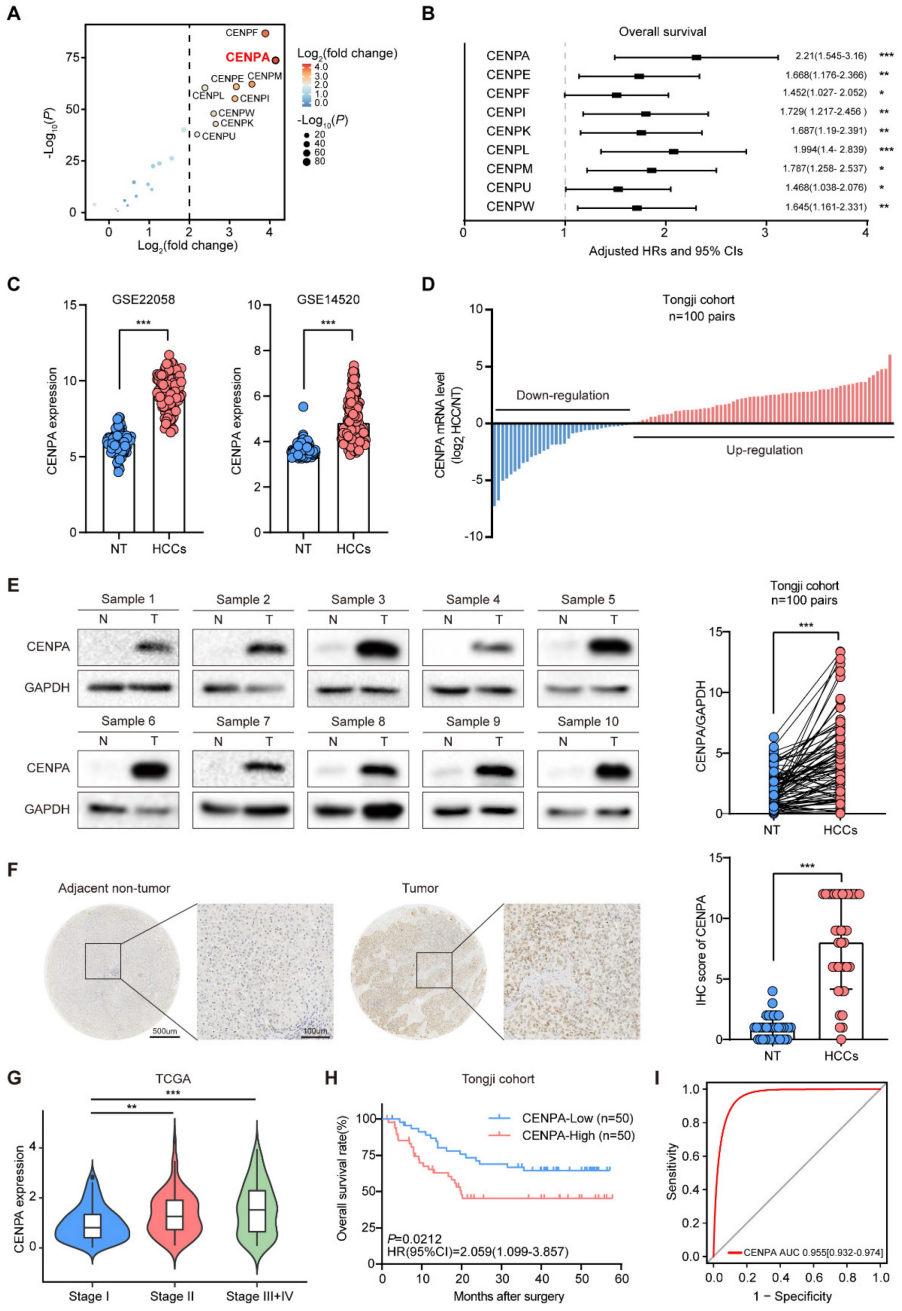
** High expression of CENPA correlates with poor outcomes in HCC patients. (A)** Comparison of CENPs family member expression between tumor tissues and normal tissues in TCGA cohort. The horizontal axis shows differences in gene expression between tumor tissues and normal tissues, and the vertical axis shows the significant of the differences. The color of the points in the figures represents the fold differences, and the size of the points represents the significance of the difference. *P* values by two-side Students *t* test. **(B)** Association of CENPs family member expression with patient OS times in TCGA cohort based on univariate cox regression analysis. HR>1 indicates that the gene is a risk factor. **(C)** CENPA mRNA expression in HCC tissues and adjacent non-tumor tissues of patients in GSE22058 and GSE14520. NT, adjacent normal tissues. HCCs, hepatocellular carcinoma tissues. **(D)** Differential expression of the CENPA mRNA in 100 paired HCC tissues and adjacent non-tumor tissues of patients in Tongji cohort as determined by qRT-PCR. **(E)** CENPA protein level in 100 paired nontumor (N) and tumor (T) liver tissues from Tongji cohort as determined by western blotting, the band intensity was calculated by Image J software (*P*<0.0001). GAPDH was used as loading control. **(F)** Representative IHC staining images of the CENPA expression in HCC and adjacent tissues in Tongji HCC TMA cohort and a statistical evaluation of the staining intensity (*P*<0.0001). Scale bars=500um or 100um, respectively. **(G)** The expression of CENPA of HCC patients classified with pathological grades in TCGA database. **(H)** Kaplan-Meier analysis of overall survival rate for HCC patients based on CENPA expression in the Tongji cohort (*P*=0.0212). **(I)** ROC curve of high-expressed CENPA to evaluate the diagnosis efficiency of HCC. Data are shown as mean ± SEM; n = 3 independent experiments. The statistical significance was determined by Student's two-tailed t test. *P < 0.05; **P < 0.01; ***P < 0.001.

To investigate the potential role of the CENPs family in HCC, we first analyzed the mRNA expression patterns of CENPs family using RNA sequencing (RNA-seq) data from The Cancer Genome Atlas (TCGA) database, with 375 liver tumor tissues and 50 adjacent tissues. Among the CENPs family genes, CENPA, CENPE, CENPF, CENPI, CENPK, CENPL, CENPM, CENPU, and CENPW significantly up-regulated in the HCC tissues compared with the adjacent tissues (Figure 1A; Table S1). Univariate cox regression analysis in the TCGA cohort demonstrated that the expression level of these nine CENPs family members were risk factors for HCC prognosis (Figure 1B; Figure S1A). Among these genes, CENPA showed higher expression and the highest hazard ratio (HR) for OS in HCC (Figure 1A and 1B; Figure S1A), therefore we focused on the role of CENPA in the progression of HCC.

We further validated the expression pattern of CENPA in GSE22058 and GSE14520 datasets and found that the expression level of CENPA in HCC was significantly higher than in adjacent non-tumor tissues (Figure 1C). Then we performed real-time quantitative PCR (qRT-PCR) and western blotting in 100 pairs HCC samples from Tongji hospital, and found that the mRNA and protein level of CENPA were up-regulated in tumor tissues compared to adjacent non-tumor tissues (Figure 1D and 1E). In addition, immunohistochemical (IHC) staining in an independent cohort of 37 paired HCC samples indicated that expression of CENPA was increased in HCC tissues compared with the adjacent non-tumor tissues (Figure 1F). In HCC cell lines profiled in the Cancer Cell Line Encyclopedia Project, we found that CENPA mRNA was generally highly expressed (Figure S1B).

We also analyzed the correlation between CENPA expression and the HCC tumor stage and found the expression level of CENPA was positively correlated with the HCC tumor stage (Figure 1G). Further analysis of the relationship between CENPA expression and prognosis showed that the patients with high CENPA mRNA expression had shorter OS than those with low expression (Figure 1H). The receiver operating characteristic (ROC) curve indicated CENPA expression level showed a high correlation with HCC diagnosis (Figure 1I). These results demonstrated that CENPA is highly expressed in HCC and highly expressed CENPA predicted poor prognosis for HCC patients.

### CENPA promotes HCC cell proliferation and tumor growth

**Figure 2:**
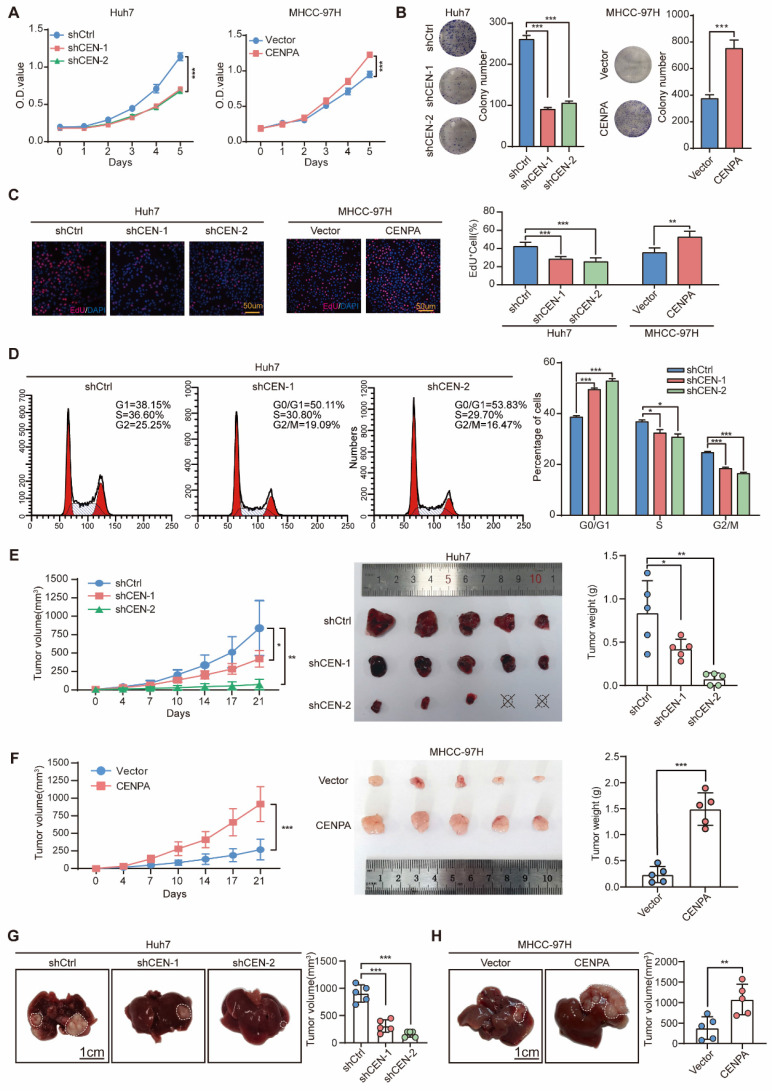
** CENPA promotes HCC cell proliferation, cell cycle progression and tumor growth *in vitro* and* in vivo.* (A)** CCK-8 assay of Huh7 and MHCC-97H cells after CENPA knockdown or overexpression. **(B)** Clone formation assays of Huh7 and MHCC-97H cells after CENPA knockdown or overexpression. **(C)** Representative images of EdU assays and quantification of EdU^+^ cells in CENPA knockdown or overexpression cells. **(D)** Statistical analysis revealed the cell cycle changes after CENPA knockdown in Huh7 cells. **(E)** Subcutaneous tumor growth assay in mice injected with CENPA knockdown Huh7 cells. Growth curves and weight of subcutaneous tumor in the indicated groups (n=5 mice/group). **(F)** Subcutaneous tumor growth assay in mice injected with CENPA overexpression in MHCC-97H cells. Growth curves and weight of subcutaneous tumor in the indicated groups (n=5 mice/group). **(G)** Representative tumor images and tumor volumes after CENPA knockdown in the orthotopic Huh7 cell model. **(H)** Representative tumor images and tumor volumes after CENPA overexpression in the orthotopic MHCC-97H cell model. Data are shown as mean ± SEM; n = 3 independent experiments. The statistical significance was determined by Student's two-tailed t test. *P < 0.05; **P < 0.01; ***P < 0.001. OD optical density.

To clarify the biological function of CENPA in HCC, both gain- and loss- of function studies were performed. We first detected the CENPA expression level, and found that compared to HL7702, CENPA was significantly upregulated in HCC cell lines (Figure S2A). Then, we constructed stable CENPA knockdown cell line in Huh7 cells, and overexpression cell lines in MHCC-97H and HLF cells (Figure S2B). After knockdown of CENPA, the proliferation and colony formation capabilities of the Huh7 cells were significantly inhibited (Figure 2A and 2B), whereas overexpression of CENPA in MHCC-97H and HLF cells enhanced the proliferation and colony formation capabilities of HCC cells (Figure 2A and 2B; Figure S2C and S2D). The EdU assay further demonstrated that CENPA was essential for cell proliferation, as knocking down CENPA indicates a pronounced reduction in DNA synthesis, and vice versa (Figure 2C). Since DNA synthesis is closely correlated with cell cycle, we examined the change of cell cycle procession by flow cytometry. The results suggested that CENPA decreased the proportion of HCC cells in the G0/G1 phase, while the number of cells in the G2/M phase was increased, and CENPA knockdown resulted in the opposite phenotypic changes (Figure 2D; Figure S2E).

We then used heterotopic xenograft and orthotopic xenograft animal models to further identify the role of CENPA in HCC cell proliferation *in vivo*. Results showed that compared with the control group, the tumor size and weight of the CENPA knockdown group were significantly reduced after subcutaneous injection (Figure 2E; Figure S2F). The opposite results were observed in the CENPA overexpressed group (Figure 2F; Figure S2G). The orthotopic xenograft tumor model showed that compared with the control group, CENPA knockdown significantly reduced the volume of orthotopic liver tumor, while the overexpression of CENPA had the opposite effect (Figure 2G and 2H; Figure S2H and S2I). IHC staining results showed that, compared with controls, the positive proportion of Ki67 was lower in tumors with CENPA knockdown but higher in tumors with CENPA overexpression (Figure S2F-S2I). All these results suggested that CENPA promotes HCC cell proliferation and tumor growth.

### CENPA interacts with YY1 and promotes its transcriptional activity

**Figure 3:**
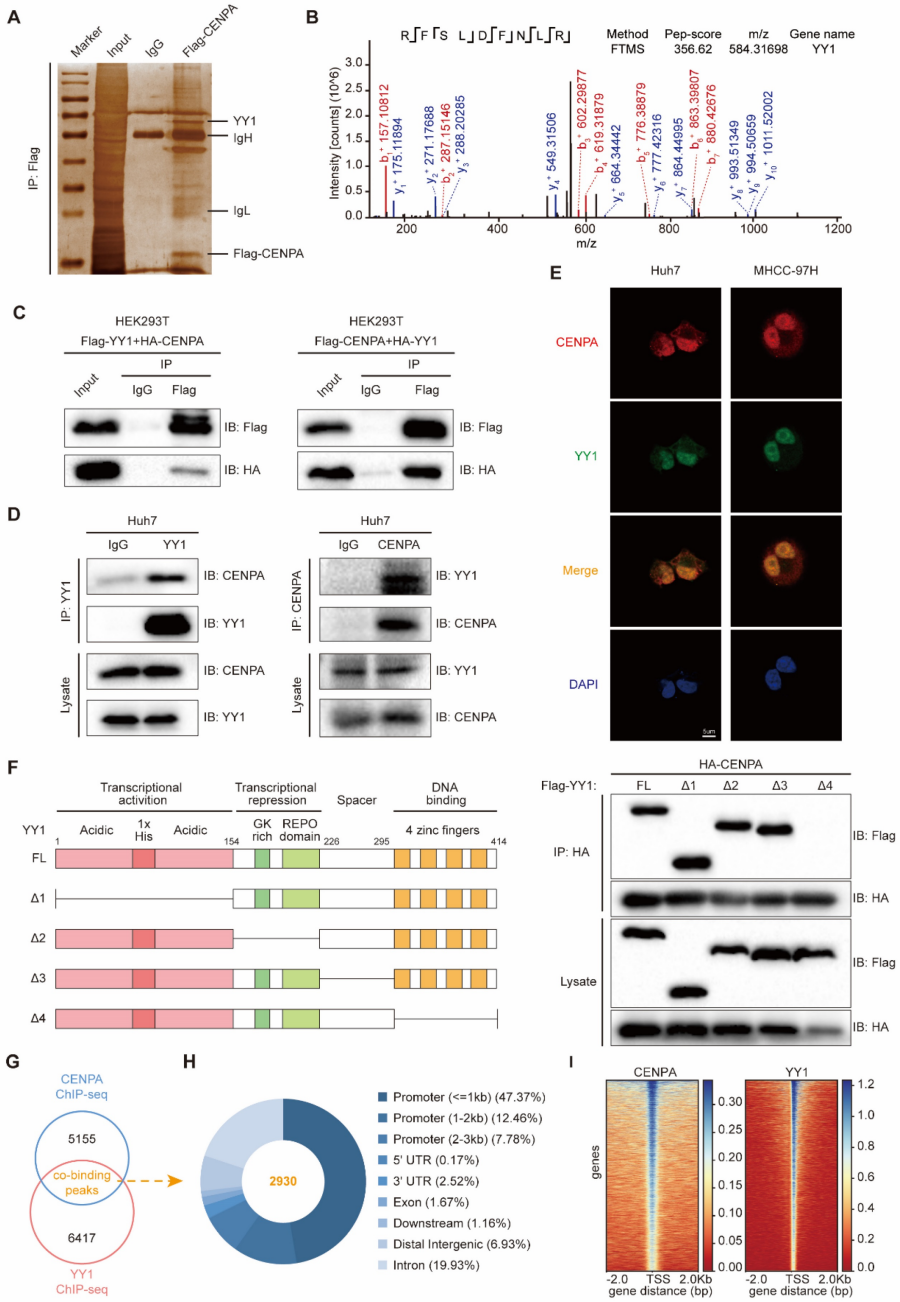
** CENPA interacts with YY1 and collaborate as co-transcriptional complex. (A)** Silver staining of protein enriched by Flag-CENPA in HEK293T cells. The arrow indicated the additional bands that were enriched by CENPA as compared to control. **(B)** The peptide fragment of YY1 as determined by mass spectrometry. **(C)** Co-IP assays were conducted in HEK293T cells transfected with a Flag-YY1 and HA-CENPA or Flag-CENPA and HA-YY1. **(D)** Endogenous co-IP assays were performed in Huh7 cells, IgG was used as control. **(E)** Confocal immunofluorescence showed that CENPA and YY1 were colocalized in the cell nucleus. Nuclei were counterstained with DAPI (Blue). Scale bar: 5um. **(F)** Scheme of YY1 domain and truncations, and co-IP assays were performed in HEK293T cells co-transfected with HA-CENPA together with a series of Flag-YY1 truncations. **(G)** Specific peaks of genomic binding were identified by CENPA and YY1 ChIP-seq in Huh7 cells. **(H)** Elements characteristics of overlapping regions were analyzed. **(I)** The distributing patterns of the ChIP-seq assays of CENPA and YY1.

To study the mechanism by which CENPA promotes HCC proliferation, we first performed the co-immunoprecipitation (co-IP) assay to enrich the proteins interacting with CENPA. By mass spectrometry analysis, we found that YY1, as one of the top candidates, interacted with CENPA (Figure 3A and 3B; Table S2). The interaction between CENPA and YY1 was confirmed by co-IP in HEK293T cells (Figure 3C). We further performed co-IP experiments with whole-cell lysate from Huh7 cells to confirm the protein-protein interaction between the endogenous CENPA and YY1 (Figure 3D). In addition, confocal microscopy experiments showed that CENPA colocalized with YY1 in HCC cells (Figure 3E). YY1 consists of transcriptional activation domain (residues 1-154), transcriptional repression domain (residues 155-226), spacer domain (residues 227-295) and DNA-binding domain (residues 296-414) 31. To identify the functional domain of YY1 that interacts with CENPA, YY1 deletion mutants (YY1 Δ1, YY1 Δ2, YY1 Δ3, YY1 Δ4) were constructed. We found that YY1 Δ4 (in which the zinc finger region was deleted) lost the ability to interact with CENPA (Figure 3F), indicating that the binding of YY1 to CENPA depended on the zinc finger region.

Since YY1 is a member of the GL-Kruppel family of zinc finger DNA binding proteins that can activate or inactivate gene expression depending on partner proteins, promoter context, and chromatin structure 32. Recent studies also reported that CENPA might function as a transcription regulator 19. Considering the previous studies, we hypothesize whether CENPA interacts with YY1 forming the co-transcription factor complex. To chart the co-binding landscape of CENPA and YY1 on a genome scale, we conducted CENPA ChIP-sequencing (ChIP-seq) and YY1 ChIP-seq in Huh7 cell. Analysis of confluence revealed that CENPA and YY1 contain 2930 co-binding sites/regions (Figure 3G). A majority of the sites/regions (~72%) were localized in transcriptional start elements, including the promoter, 5'-UTR, and 1st exon, followed by the distal intergenic region (6.93%) (Figure 3H). Of those sites/regions, similar distribution patterns in CENPA and YY1 (Figure 3I) and a closer spatial distance between the binding tracks were observed (Figure S3A). Since transcription factors exert transcriptional regulatory functions by directly binding to DNA region of chromatin. To investigate the chromatin accessibility of transcription factors CENPA and YY1, we conducted the chromatin isolation assay 33. The chromatin isolation assay in CENPA overexpression or YY1 knockdown in MHCC-97H cells showed that both CENPA and YY1 directly bound to the chromatin (Figure S3B and S3C). In addition, CENPA enhanced the chromatin accessibility of YY1 (Figure S3B). Additionally, KEGG analysis showed that compared to CENPA or YY1 alone (Figure S3D-S3E), these genes co-regulated by CENPA+YY1 are specially enriched in some tumor-related signaling pathway such as cell cycle and cellular senescence (Figure S3F). Thus, we demonstrated that CENPA interacts with YY1, forming the transcriptional complex, to co-regulate a set of genes involved in tumor progression.

### YY1 is essential for CENPA-mediated HCC progression

**Figure 4:**
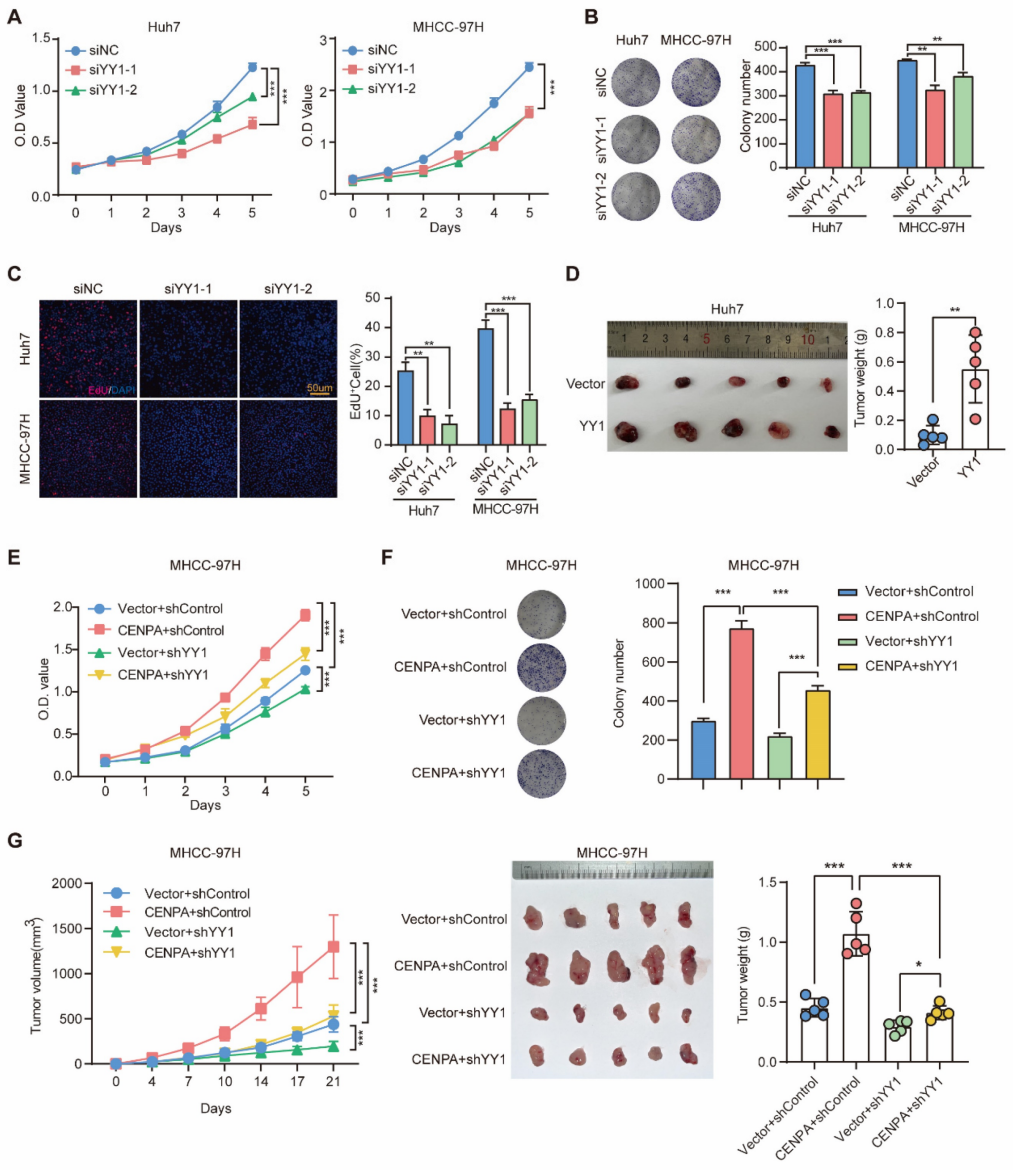
** YY1 mediate CENPA to regulate HCC progression. (A)** CCK-8 assay of Huh7 and MHCC-97H cells after YY1 knockdown cells. **(B)** Clone formation assays of Huh7 and MHCC-97H cells after YY1 knockdown cells. **(C)** Representative images of EdU assays and quantification of EdU^+^ cells in YY1 knockdown cells. **(D)** Subcutaneous tumor growth assay in mice injected with Huh7 YY1 overexpression cells. Weight of subcutaneous tumor in the indicated groups (n=5 mice/group). **(E)** CCK-8 assay of YY1 knockdown on the basis of CENPA overexpression MHCC-97H cells. **(F)** Clone formation assays of YY1 knockdown on the basis of CENPA overexpression MHCC-97H cells. **(G)** Subcutaneous tumor growth assay in mice injected with YY1 knockdown on the basis of CENPA overexpression MHCC-97H cells. Growth curves and weight of subcutaneous tumor in the indicated groups (n=5 mice/group). The statistical significance was determined by Student's two-tailed t test. *P < 0.05; **P < 0.01; ***P < 0.001.

As a transcription factor, YY1 plays the dual role as transcriptional activator and repressor in different tumors 34. To investigate the biological function of YY1 in HCC, we silenced or overexpressed the expression of YY1 in Huh7 and MHCC-97H cells (Figure S4A and S4B). *In vitro* experiments with HCC cell lines showed that knockdown of YY1 reduced cell viability and clonogenic capacity (Figure 4A-4C) and vice versa (Figure S4C). Consistent results were obtained *in vivo* with subcutaneous tumors (Figure 4D; Figure S4D).

To explore whether the oncogenic role of CENPA was through YY1, we constructed YY1 knockdown cell line in CENPA overexpression MHCC-97H cell (Figure S4E). YY1 knockdown significantly weakened the effect of CENPA overexpression on promoting HCC cell proliferation *in vitro* (Figure 4E and 4F). Similar results were observed *in vivo* with subcutaneous tumors (Figure 4G and Figure S4F). Therefore, these results suggest that YY1 acts as an essential mediator of CENPA to regulate the progression of HCC.

### CCND1 and NRP2 may serve as the key candidate genes regulated by CENPA and YY1

**Figure 5:**
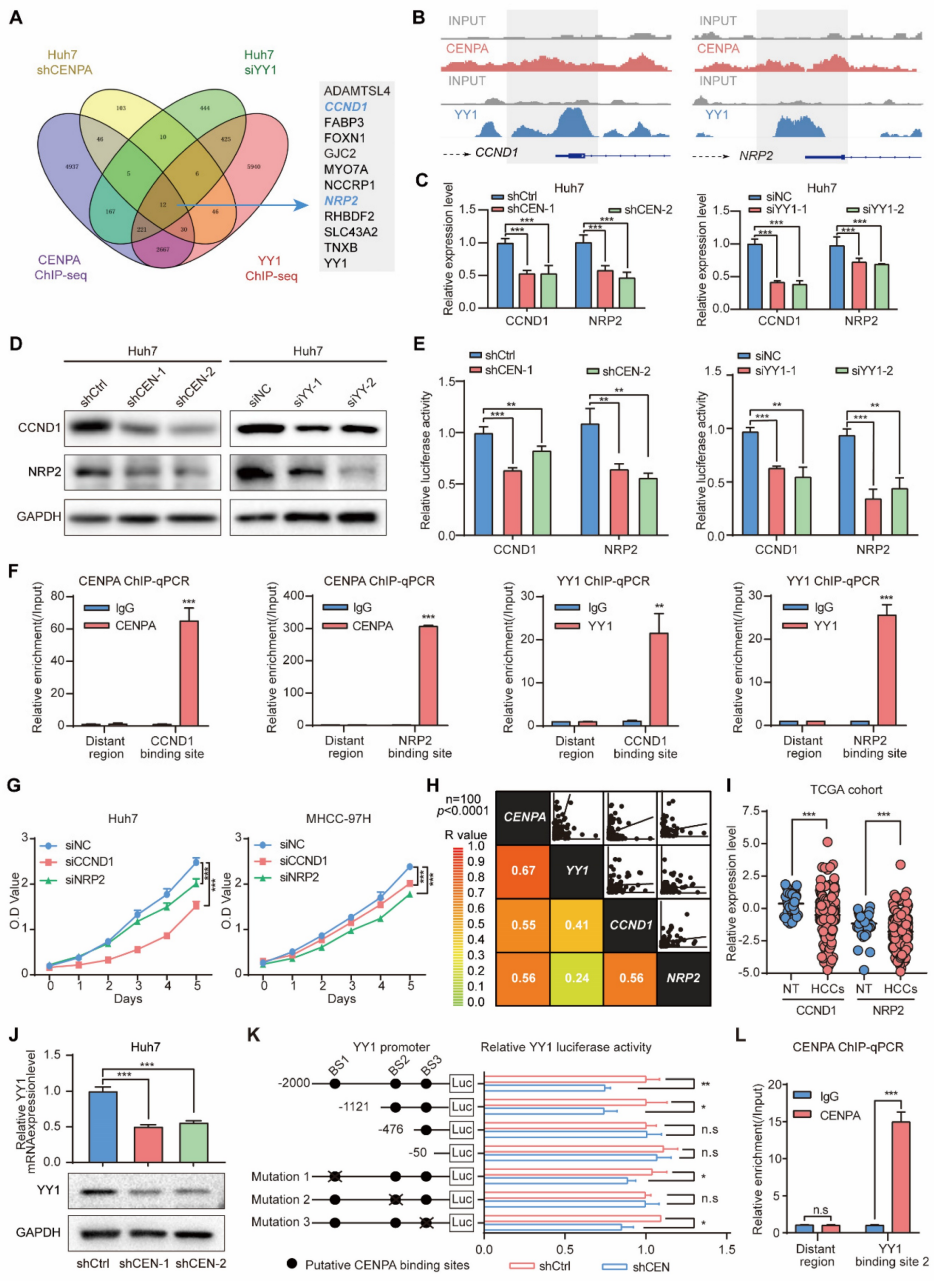
** CCND1 and NRP2 are oncogenes induced by CNEPA /YY1 complex. (A)** Positively regulated genes were identified by RNA-seq in Huh7 cells of CENPA or YY1 knockdown and ChIP-seq in Huh7 of CENPA or YY1, in which 12 co-regulated genes were showed. **(B)** Binding tracks of CENPA or YY1 on the TSS region of CCND1 and NRP2, input was used as negative control. **(C)** qRT-PCR detection of mRNA level of CCND1 and NRP2 in Huh7 of CENPA or YY1 knockdown, respectively. **(D)** Western blotting detection of protein level of CCND1 and NRP2 in Huh7 of CENPA or YY1 knockdown, respectively. **(E)** Luciferase assay identified CENPA and YY1 promote the transcription activity of CCND1 and NRP2. **(F)** ChIP-qPCR assays demonstrated the binding of CENPA and YY1 at the promoter regions of CCND1 and NRP2. **(G)** CCK-8 assay of Huh7 and MHCC-97H cells after CCND1 or NRP2 knockdown cells. **(H)** Correlation analysis of mRNA expression among CENPA, YY1, CCND1 and NRP2 in Tongji cohort (*p*<0.0001). **(I)** CCND1 and NRP2 mRNA expression in HCC tissues and adjacent non-tumor tissues of patients in TCGA database. NT, adjacent normal tissues. HCCs, hepatocellular carcinoma tissues. **(J)** qRT-PCR and western blotting detection of expression level of YY1 in Huh7 CENPA knockdown cells. **(K)** Schematic diagram of the 3 putative CENPA-binding sites located in the promoter region of YY1 and the pGL4.17 based YY1 promoter reporter constructs. The transcription activity of the luciferase reporter constructs with WT and mutant CENPA-binding sites were determined by luciferase assay. **(L)** The binding between CENPA and putative binding sites in YY1 promoter was determined by ChIP assay. The statistical significance was determined by Student's two-tailed t test. *P < 0.05; **P < 0.01; ***P < 0.001.

To further investigate the downstream genes involved in the mechanism through which CENPA and YY1 collaborate to promote HCC proliferation, we conducted RNA-seq in Huh7 following knockdown CENPA or YY1 expression. Integrative analysis of transcriptome and ChIP-seq data revealed that 12 genes were potentially highly related direct target genes that down-regulated in HCC cells after knockdown of CENPA and YY1 (Figure 5A). We further confirmed that CCND1 and NRP2 were the main target genes of CENPA/YY1 complex by qRT-PCR in Huh7 and MHCC-97H cells (Figure S5A). With further research, we identified co-binding tracks of CENPA and YY1 on the promoters of CCND1 and NRP2 (Figure 5B). The qRT-PCR and western blotting assays further confirmed that CCND1 and NRP2 were positively regulated by both CENPA and YY1 (Figure 5C and 5D; Figure S5B). Luciferase and ChIP-qPCR analyses demonstrated that CENPA and YY1 promoted the transcription efficiency of CCND1 and NRP2 via binding at their promoter regions (Figure 5E and 5F). Moreover, YY1 cannot bind to the CCND1 and NRP2 promoter regions after losing its interaction domain (YY1 Δ4) with CENPA (Figure S5C). These results validated that CCND1 and NRP2 serve as the key candidate genes of the CENPA/YY1 transcription complex in HCC cells.

We further investigated the biological effect of CCND1 and NRP2 in HCC cell lines. Functionally, the knockdown of CCND1 and NRP2 suppressed the HCC cell growth of Huh7 and MHCC-97H (Figure 5G; Figure S5D-S5G). Clinically, correlation analysis of integrated mRNA data from Tongji cohort 100 pairs HCC patients revealed significant positive correlations among the expression of CENPA, YY1, CCND1, and NRP2 (Figure 5H). Based on the TCGA database, we found that CCND1 and NRP2 mRNA expression was significantly increased in the HCC compared with adjacent non-tumor (Figure 5I). Collectively, these findings suggest that CENPA and YY1 collaborate to promote HCC cell proliferation via transcriptional activation of CCND1 and NRP2.

Besides CCND1 and NRP2, YY1 was also a potential target gene that down-regulated in HCC cells after the knockdown of CENPA (Figure 5A). To detect whether CENPA also regulated YY1, we employed qRT-PCR and western blotting for experimental validation, and found that knockdown of CENPA reduced the expression of YY1 at both mRNA and protein level and vice versa (Figure 5J; Figure S5H). Furthermore, the luciferase reporter assay results showed that CENPA knockdown reduced YY1 promoter activity, indicating that CENPA regulates YY1 expression at the transcriptional level (Figure 5K). Sequence analysis revealed the presence of three putative CENPA-binding sites in the YY1 promoter. Serial deletion and site-directed mutagenesis showed that the second CENPA-binding site is crucial for CENPA-induced YY1 transactivation (Figure 5K). ChIP-qPCR assay results further confirmed the direct binding of CENPA to the YY1 promoter in HCC cells (Figure 5L). These results further confirmed that YY1 was the target gene of CENPA and played a crucial role in the process of regulating HCC proliferation by CENPA.

### The K124 lactylation enhances CENPA transcriptional activity

**Figure 6:**
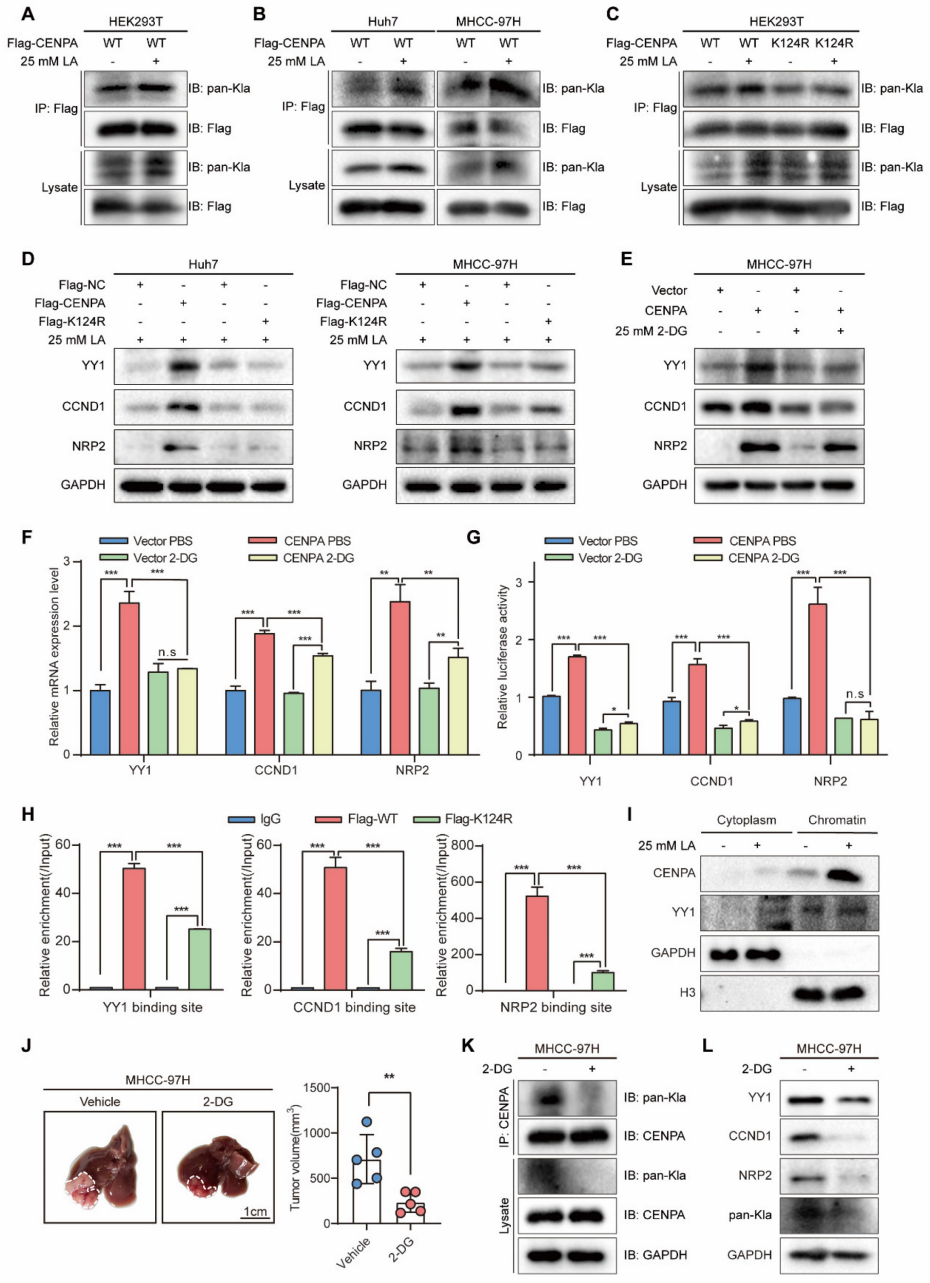
** Lactylation of CENPA enhanced the transactivation of target genes in HCC. (A)** Lactylation of CENPA in HEK293T cells was confirmed by IP method. HEK293T cells transfected with Flag-CENPA, treated with PBS or 25 mM L-lactic acid for 24h. **(B)** Lactylation of CENPA in Huh7 and MHCC-97H cells was confirmed by IP method. Huh7 and MHCC-97H cells transfected with Flag-CENPA, treated with PBS or 25 mM 2-DG for 24h. **(C)** Flag-CENPA (WT), Flag-CENPA (K124R) were transfected into HEK293T cells for 24 h. Then the indicated cells were treated with PBS or L-lactic acid for 24 h. **(D)** Flag-NC, Flag-CENPA (WT), Flag-CENPA (K124R) were transfected into Huh7 and MHCC-97H cells for 24 h. Then cells were treated with L-lactic acid for 24 h. **(E)** MHCC-97H vector and CENPA overexpression cells were treated with PBS or 25mM 2-DG for 24 h. Western blotting was used to detect the expression level of YY1, CCND1 and NRP2. **(F)** MHCC-97H vector and CENPA overexpression cells were treated with PBS or 25mM 2-DG for 24 h. qRT-PCR was used to detect the expression level of YY1, CCND1 and NRP2. **(G)** MHCC-97H vector and CENPA overexpression cells were treated with PBS or 25mM 2-DG for 24 h. Luciferase assay identified CENPA promotes the transcription activity of YY1, CCND1 and NRP2. **(H)** Flag-NC, Flag-CENPA (WT), Flag-CENPA (K124R) were transfected into Huh7 cells for 24 h. Then cells were treated with 25mM L-lactic acid for 24 h. ChIP-qPCR assays demonstrated the binding of Flag-CENPA and Flag-CENPA (K124R) at the promoter regions of YY1, CCND1 and NRP2. **(I)** After treated with PBS or 25mM L-lactic acid for 24 h, cellular fraction of MHCC-97H was performed to detect the abundance of CENPA and YY1 in cytoplasm and chromatin. **(J)** Representative tumor images and tumor volumes after treated with PBS or 25mM 2-DG for one month in the orthotopic MHCC-97H cell model. **(K)** Lactylation level of CENPA in MHCC-97H tumor tissues after treated with PBS or 25 mM 2-DG.** (L)** Expression of YY1, CCND1 and NRP2 in MHCC-97H tumor tissues after treated with PBS or 25 mM 2-DG. The statistical significance was determined by Student's two-tailed t test. *P < 0.05; **P < 0.01; ***P < 0.001.

We continued to explore the potential mechanisms responsible for the role of CENPA in HCC cells. PTMs of CENPA play vital roles in maintaining homeostasis. For example, ubiquitylation has been reported to be essential for CENPA deposition at the centromere 23, 35. Recently, lactylation is a novel PTM of histone proteins, which has been proven tightly correlated with HCC progression 36, 37, such as activating transcription and promoting cell reprogramming 38. Moreover, recent studies revealed that inhibiting histone lactate may inhibit tumorigenesis, providing us with the potential for HCC clinical therapy 39. In addition, there have been no previous studies on the lactylation modification of CENPA. Therefore, we are interested in exploring the lactylation modification that modulates the function of CENPA. To verify whether CENPA can be lactylated in cells, immunoprecipitation (IP) test was conducted, which showed that pan-Kla was indeed present in CENPA, and lactic acid (LA) increased the lactylation level of CENPA (Figure 6A). Moreover, CENPA lactylation also can be detected in HCC cell lines, which could be enhanced by 25-mM LA (Figure 6B). Collectively, these data suggested that lactylation modification did exist on CENPA.

Previous studies have shown that lysine lactylation (Kla) is a new type of histone mark 29, 36. Among the 7 lysine amino acids present in the human CENPA protein, CENPA-K124 contains various types of PTM, such as phosphorylation and ubiquitylation 23, 40. In addition, researches on CENPA related PTM sites suggests that K124 is crucial for cell progression 27, 41. Thus, we constructed Flag-CENPA K124 mutation (Flag-K124R) plasmid. Flag-CENPA and Flag-K124R were transfected into HEK293T cells, cell lysis was for IP with anti-Flag antibody, followed by western blotting with the anti-pan-Kla antibody, and the results showed that K124R of CENPA extensively diminished the overall lactylation level of CENPA (Figure 6C).

Then we speculated whether the K124 lactylation of CENPA enhances its transcriptional activity. After transfecting Flag-NC, Flag-CENPA, and Flag-K124R with 25 mM LA into HCC cell lines, we identified the target genes of CENPA. Compared with Flag-CENPA, the expression of CENPA was significantly reduced by transfection with Flag-K124R (Figure 6D). Likewise, 2-deoxy-D-glucose (2-DG), a non-metabolizable glucose analog, significantly reduced the effect of CENPA overexpression on promoting the mRNA and protein expression level of its target genes in HCC cells (Figure 6E and Figure 6F). Luciferase assay showed the same results that 2-DG reduced the effect of CENPA overexpression on promoting the transcription activity of its target genes (Figure 6G). Additionally, CENPA-K124R mutation markedly reduced the binding of CENPA to the promoters of YY1, CCND1, and NRP2 in Huh7 cell by ChIP-qPCR (Figure 6H). To detect whether lactic acid enhances the chromatin accessibility of CENPA, chromatin isolation assay was conducted. Result indicated that lactylated CENPA was more likely to bind to chromatin (Figure 6I). To further explore the role of CENPA lactylation *in vivo*, an orthotopic xenograft animal models was established. The treatment with 2-DG slowed down the growth of MHCC-97H tumors (Figure 6J). In addition, we detected a decrease in CENPA lactylation level and downstream genes protein level (such as YY1, CCND1 and NRP2), in 2-DG treated HCC tumor tissues (Figure 6K and 6L). These results indicate that the lactylation of CENPA K124 enhanced its transcriptional activation ability in HCC cells and facilitate HCC progression.

### CENPA expression is positively correlated with YY1 expression in human HCC tissues

**Figure 7:**
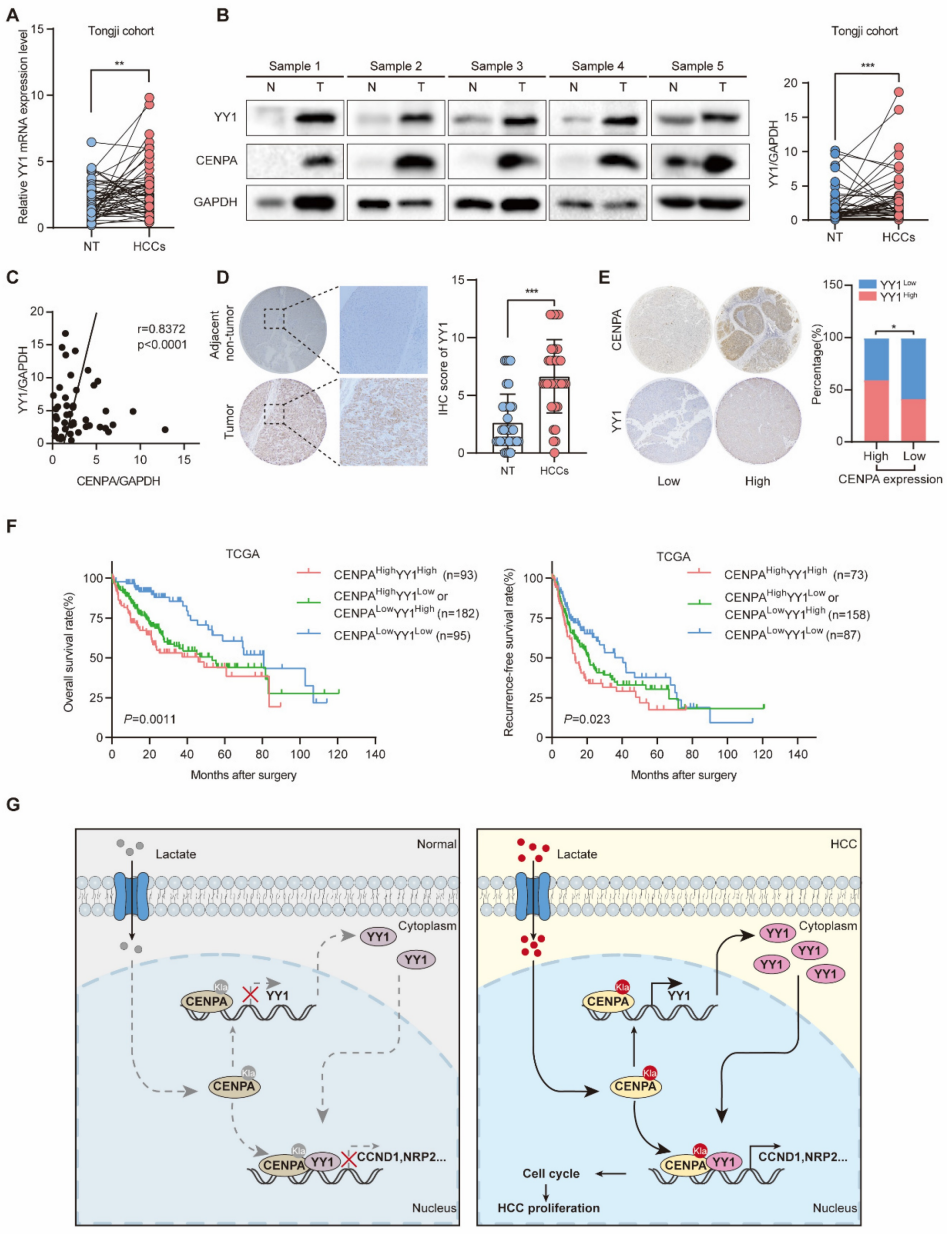
** CENPA expression is positively correlated with YY1 expression in human HCC tissues. (A)** YY1 mRNA expression in HCC tissues and adjacent non-tumor tissues of paired patients in Tongji cohort. **(B)** YY1 protein level in nontumor and HCC tissues from Tongji cohort as determined by western blotting, the band intensity was calculated by Image J software (*P*<0.001). GAPDH was used as loading control. **(C)** The correlation analysis of the CENPA and YY1 protein levels in Tongji cohort as determined by qRT-PCR. **(D)** Representative IHC staining images of the YY1 expression in HCC and adjacent tissues in Tongji HCC TMA cohort and a statistical evaluation of the staining intensity (*P*<0.0001). **(E)** The IHC scores demonstrated a high correlation between CENPA and YY1 staining in Tongji HCC TMA cohort (chi-square test). **(F)** Kaplan-Meier curves of the OS and RFS rates among groups with differential CENPA/YY1 expression in TCGA database. **(G)** A schematic model of the mechanism by which CENPA promotes HCC progression. The statistical significance was determined by Student's two-tailed t test. *P < 0.05; **P < 0.01; ***P < 0.001.

To determine the relevance in HCC, we performed western blotting and IHC staining to examine the expression of YY1 and CENPA. In Tongji HCC samples, YY1 was highly expressed in HCC as determined by qRT-PCR and western blotting (Figure 7A and 7B). The correlation analysis showed that CENPA was strongly positively correlated with YY1 in HCC tissues (Figure 7C). Consistent result was obtained by IHC staining (Figure 7D). Moreover, HCC patients with high CENPA expression were more likely to have high expression of YY1 than those with low CENPA expression (chi-square, *P*=0.0105, Figure 7E). In addition, we analyzed the prognostic significance of CENPA and YY1 expression in HCC patients. Patients with high expression of both CENPA and YY1 had the worst OS and RFS rates among all patients analyzed (Figure 7F). These results indicate that both CENPA and YY1 are highly expressed in HCC and have similar expression characteristic. High expression of both CENPA and YY1 suggests a poor prognosis in HCC patients.

## Discussion

In this study, we found that CENPA interacted with YY1 and formed the CENPA/YY1 transcription complex. RNA-seq and ChIP assays indicated that the CENPA/YY1 complex occupied many target genes promoters and was associated with increased transcription efficiency as a transcriptional activator. Lactation modification of the CENPA K124 site could enhance the transcription activation function of CENPA. Functional experiments demonstrated that CENPA/YY1 complex could promote HCC cell proliferation by activating the expression of CCND1 and NRP2 in HCC (Figure. 7G).

CENPs are highly conserved centromere-related proteins that control the balance of chromosome segregation. CENPs family is highly expressed in many cancers, but its functions in HCC have yet to be fully clarified. Thus, a better understanding of the biological functions of CENPs is essential to understanding the proliferation and development of HCC. In this study, we identified that the increased expression of CENPA was correlated with poor prognosis of HCC patients and was characterized as an independent prognostic factor in our HCC cohort. CENPA has previously been discovered to play a role in malignant tumor progression. For example, CENPA can accelerate the cell cycle by triggering the Wnt/β-catenin pathway in clear cell renal cell carcinoma (ccRCC) cells 42. In addition, researchers demonstrated that CENPA promotes the expression of Myc in retinoblastoma 43. However, its functional effects, especially the mechanism of mediating the proliferation and growth in HCC cells, were largely unknown. Here, we presented novel research that CENPA play its role as an oncogene in HCC. Moreover, CENPA formed the transcriptional complex with YY1 to up-regulate the transcription efficiency of their target genes, suggesting the new role of CENPA that functions as the histone H3 variant. Although more evidence is needed to further support this new transcription complex, our study reveals the potential function of CENPA in tumor progression.

YY1 was reported to regulate many crucial biological and cellular processes, including transcription, DNA repair, DNA replication, cell proliferation and differentiation, and embryogenesis 44, 45. Most cancer-related processes are mediated by YY1, which strongly implicates the importance of YY1 in cancer development and progression 46, 47. Indeed, our data demonstrated that YY1 could interact with CENPA and form transcription complexes. Moreover, overexpression of YY1 has been observed in various types of cancer. Here, we presented the oncogene role of YY1, which acted as a transcription factor in HCC.

Furthermore, our results indicated that YY1 was also positively regulated by CENPA via the binding of its promoter region, which further highlights the vital role of YY1 in the carcinogenesis of CENPA. Clinically, CENPA and YY1 are positively correlated with each other in HCC patients. Combined with our results, CENPA and YY1 may be potential therapeutic targets for HCC progression.

PTM of CENPA plays an essential role in various nuclear processes, including transcriptional activation, silencing, and mitosis 48, 49. In this study, we identified the novel PTM type of CENPA at the K124 site. Previous studies revealed that increased lactylation of histones in the promoter regions had been proven to induce the expression of homeostatic genes. Here, we demonstrated that CENPA lactylation might lead to oncogene expression and accelerate the tumorigenesis of HCC. In our study, we presented that lactylated CENPA can promote its binding at YY1, CCND1 and NRP2 promoter regions and enhance transcription efficiency. However, it is unclear whether this CENPA lactylation site has more essential functions, which needs further study.

## Conclusion

In conclusion, through an expression level and clinical correlation screen of CENPs family members in HCC, CENPA was found to be a potential risk factor for HCC. CENPA is highly expressed in HCC tissues and is correlated with a poor prognosis in HCC patients. Mechanistically, CENPA promotes the growth of HCC by interacting with YY1 and forming the CENPA/YY1 complex. Then, the CENPA/YY1 complex drives HCC proliferation via promoting the transcription activity of CCND1, NRP2 and YY1. Moreover, the lactylation of CENPA enhanced its transcription efficiency. The findings in the present study led us to expect that targeting CENPA could be a potential treatment for HCC.

## Supplementary Material

Supplementary materials and methods, figures and tables.

## Abbreviations

HCC: Hepatocellular carcinoma
CENPs: Centromere proteins
CENPA: Centromere protein A
CCND1: Cyclin D1
NRP2: Neuropilin 2
OS: Overall survival
CCAN: Constitutive Centromere-Associated Network
PTM: Post-transcriptional modification
RFS: Recurrence free survival
HR: Hazard ratio
LA: Lactic acid
2-DG: 2-deoxy-D-glucose
