# Supplementary material for: CENPA functions as a transcriptional regulator to promote hepatocellular carcinoma progression via cooperating with YY1
Source: Int J Biol Sci. 2023 Oct 16;19(16):5218–32. doi: 10.7150/ijbs.85656 (PMC10620822; doi:10.7150/ijbs.85656)
Supplement: Supplementary file 1 — Supplementary materials and methods, figures and tables. [file ijbsv19p5218s1.pdf]

Supplementary information

Supplementary Materials and Methods

Quantitative real-time PCR

Total RNA was extracted from cells or human tissues using TRIzol Reagent (Invitrogen, USA) according to the manufacturer’s instruction. Then 1 μg of total RNA was used to synthesize cDNA using HiScript III 1st Strand cDNA Synthesis Kit (Vazyme Biotech co.,ltd). The cDNA applied to real-time PCR analysis with specific primers and ChamQ SYBR Color qPCR Master Mix (Vazyme Biotech co.,ltd) utilizing a CFX Connect™ Real-Time PCR Detection System (Bio-Rad, Hercules, CA, USA) in accordance with the manufacturer’s instructions. Each experiment was performed three times independently, and the $2^{-\Delta\Delta Ct}$ method was used to analyze the results. GAPDH was used as endogenous control.

Western blotting

Cell or human tissues were lysed in RIPA buffer mixed with EDTA-free protease inhibitor cocktail and a phosphatase inhibitor cocktail (Roche). Next, measuring the protein concentrations by BCA assay. 40μg of total protein were loaded and separated using 10% sodium dodecyl sulfate-polyacrylamide gels electrophoresis (SDS-PAGE), and then transferred onto polyvinylidene fluoride (PVDF) membranes (Immobilon-P; Millipore Corporation, MA, USA). Then, block the membranes in 5% skim milk diluted by Tris-buffered saline/Tween (TBST) for 1 h, and incubated with the indicated primary antibodies at 4 °C overnight. The membranes were then incubated with the secondary antibody at room temperature for 1 h. The protein bands were visualized using

Clarity^{TM} Western ECL substrate (Bio-Rad, USA) and Bio-Rad GelDoc system (Bio-Rad, USA). In Table S3, relevant antibodies' specific details were listed in.

Immunohistochemistry (IHC)

Immunohistochemistry staining for tissues was performed by using the polymer HRP detection system (Zhongshan Goldenbridge Biotechnology). The tissue slices' paraffin sections underwent dewaxing, antigen retrieval with 0.01 M sodium citrate buffer (pH 6.0), incubation with 3% H_2O_2 at room temperature for 15 min to lessen non-specific staining, and then blocking with 5% bovine serum albumin for 60 min. Then incubated with the relevent primary antibodies overnight at 4℃ in a humidified environment, followed by a 45 min incubation with HRP conjugated secondary antibody at room temperature. The antibody binding was then detected using DAB, and the cells were counterstained with hematoxylin. For each IHC test, appropriate positive and negative controls were provided. The staining intensity and proportion of positive stained tumor cells were used to score the immunohistochemical staining. The staining intensity scoring rules were as follows: 0 points (Negative); 1 point (Light brown); 2 points (Brown); 3 points (Dark brown). The stained positive cells scoring rules were as follows: score 0 denotes less than 10%, score 1 denotes 10~25%, score 2 denotes 26~50%, score 3 denotes 51~75% and score 4 denotes more than 75% of positive stained tumor cells. A total score of <6 and ≥ 6 was defined as negative and positive, respectively.

Cell culture

The normal hepatocyte cell line (L-02) and HCC cell line MHCC-97H, Huh7, HLF and HEK293T were purchased from China Center for Type Culture Collection (CCTCC, Wuhan, China). In a humidified 5% CO2 and 37 °C incubator, all cell lines were grown in the Dulbecco's modified Eagle medium (DMEM) (Hyclone, UT, USA) with 10% fetal bovine serum (Gibco, Grand Island, NY, USA).

Plasmid construction, transfection, plasmid and siRNA

The full-length CDSs of CENPA and YY1 were purchased from Addgene. Wuhan Tsingke Company synthesized the K124R site mutation plasmid of CENPA. CENPA and YY1 were ligated into the expression vectors pcDNA3.1, pcDNA3.1-Flag, and pcDNA3.1-HA, respectively. For HEK293T cells, transfection with plasmids was performed using polyethyleneimine (PEI, Invitrogen, USA) at a plasmid: PEI ratio of 1: 4. For HCC cell lines, transfection with plasmids was performed using Lipofectamine 3000 (Thermo Fisher) and P3000 (Thermo Fisher) at a plasmid: Lipofectamine 3000: p3000 ratio of 1: 1.5: 2. HCC cell lines were transfected with siRNA at a final concentration of 20nM using Lipofectamine 3000 (Thermo Fisher) according to the manufacturer’s instruction. As a control, a negative control siRNA (NC) was used. The transfection efficiency was assessed 48 hours after transfection using a western blotting test. The sequences of siRNA are listed in Table S3.

Lentivirus-transduced stable cell lines

In order to construct gene over-expression or knockdown cells lines, we cloned the

CDS region of CENPA into pLenti-CMV-Puro plasmid (Addgene #17448) or two short hairpin RNA (shRNA) against CENPA or a scramble sequence (Table S3) into the pLKO.1-TRC vector (Addgene #10879). Transient co-transfection of HEK293T cells with a lentiviral transducing vector and packaging vectors pMD2.G and psPAX2 was used to construct a recombinant lentivirus (lentivirus vector (8μg), pMD2.g (2.8μg), psPAX2 (9μg) and PEI (80μL)). Viral supernatant was then collected 48 hours after transfection. After co-cultured with lentivirus supernatant for 24 hours, HCC cells were selected and enriched by puromycin treatment at a final concentration of 10μg/ml in a culture medium. The effectiveness of CENPA overexpression or knockdown in HCC cells was assessed by western blotting assay.

EdU incorporation assay

The capacity of cells to replicate DNA was tested using a Cell-Light™ EdU Apollo567 *In Vitro* Imaging Kit (Ribobio, Guangzhou, China) according to the manufacturer's instructions. Briefly, the same amounts of HCC cells were seeded in 96-well plate (10000 cells/well). After 12h, the media was withdrawn and 50μM EdU solutions (diluted in DMEM) were given to the cells, which were then incubated at 37°C for 2h. After removing the EdU solutions and washing 1~2 times, the cells then fixed with 4% paraformaldehyde and incubated with 0.5% TritonX-100 for 5min. The ells were then stained for 30 min with 100μl 1x Apollo solution and the nucleus with DAPI. Image was captured with EVOS FL auto imaging system (Life Technologies, USA).

Cell counting kit-8 (CCK-8) method

The viability of the cells was determined using a CCK-8 assay kit (CCK-8, Wuhan Promoter Biological CO., LTD. #P5090). For 24h, cells were grown in 96-well plates at 1000-1500 cells per well. The media was then with draw, and the cells were grown for 1 hour at 37°C with a 10% CCK-8 solution (diluted in serum-free DMEM). The optical density (OD) value of the cell was determined, and the cell proliferation curve was constructed by measuring the 450 nm absorbance at each indicated time point.

Colony formation assays

For the colony formation assay, HCC cells were seeded in 6-well plates at a density of 1000-2000 cells per well. The medium was refreshed every 3 days. After 2 weeks, the colonies were washed in phosphate-buffered saline (PBS), fixed with 4% paraformaldehyde, stained with 0.1% crystal violet and imaged using an optical microscope.

Cell cycle assay

HCC cells were seeded in 6-well plates, after reaching the logarithmic stage of growth. HCC cells were digested and washed. The cells were resuspended in 75% ethanol for 24 h at 4°C, then the DNA was stained with propidium iodide (PI) at room temperature for 30 min in the dark. The indicated cell cycle phase of HCC cells was analyzed by flow cytometry (BD FACS Calibur, BD Biosciences, San Diego, CA,

USA).

Animal experiments

The entire protocol was carried out in accordance with the "Guide for the Care and Use of Laboratory Animals" (NIH publication 86-23, revised 1985) and was authorized by Tongji Hospital's Committee on the Ethics of Animal Experiments (TJH-20210312). Male BALB/C nude mice (4 weeks old) were purchased from Beijing HFK Bioscience Co. Ltd. (China) and were kept in a specific-pathogen-free (SPF) environment. In the subcutaneous tumor formation assay, we injected 1×10^6 HCC cells in 100μl of serum-free DMED subcutaneously into the flanks of nude mice (5 mice per group). The tumor volumes were assessed every 3 days, and tumor weight was recorded after 4-8 weeks of sacrifice. The volume was calculated according to below: (volume, mm^3) = 0.5 × L (length, mm) $\times W^2$ (width, mm^2). To evaluate the tumor growth *in vivo*, we injected 1×10^6 HCC cells diluted 30μl serum-free DMEM into the nude mice livers (5 mice per group). 4 weeks later after transplantation, harvested all mice tumor. Tumor volumes and tumor weights were measured as indicated above. In the tumor formation assay, we injected 1×10^6 HCC cells in 30μl of serum-free DMED subcutaneously into the liver of mice (5 mice per group), and tumor weight was recorded after 4 weeks of sacrifice. The 2-DG group, mice were received intraperitoneal injection of 400mg/kg every 3 days.

Co-immunoprecipitation (Co-IP), Liquid chromatography-tandem mass spectrometry (LC-MS/ MS) analysis, silver staining

Harvested HCC cells were suspended in 1 ml IP-lysis buffer (50 mM Tris-HCl, 150 mM NaCl, 1% Triton X-100, 1 mM EDTA, 10% glycerol, and protease inhibitor cocktail, pH 7.4). The supernatant was subjected to ultrasonication, and centrifugation was performed at 12000g for 15 min at 4°C. After that, Protein G agarose beads were used to preabsorbed the lysates of cells without or with stable transfection of tagged constructs for 2h at 4°C. Then, incubated the supernatant with the target antibody or IgG overnight in a 4°C shaker. The second day, Protein G agarose beads were added to the antibody/supernatant complex for 2 hours at 4°C. After extensive washing 5-6 times with IP-wash buffer (50 mM Tris-Cl, 300 mM NaCl, 1% Triton X-100, 1 mM EDTA, pH7.4), the samples were analyzed using western blotting to identify the potential interacting proteins. The products of co-IP assays were sent to NOVOGENE Company Limited (Beijing, China) for protein peptide detection. A Silver Stain Kit (Beyotime, Shanghai) was used to detect the expression of CENPA and its interacting proteins.

Immunofluorescence (IF)

Wild-type HCC cell lines were seeded in 24-well plate containing glass slides for 12 h. The cells were then fixed with 4% paraformaldehyde for 15 minutes, permeabilized with 0.5% TritonX-100 for 5 minutes at room temperature, and sealed in PBS containing 5% BSA. The primary antibodies were added overnight at 4°C. Cells were incubated with secondary antibodies from different species for 1 h at room temperature. Then use DAPI to counterstained nuclei for 5 min at room temperature. A Zeiss LSM 710 confocal microscope equipment was used to capture images using confocal laser-

scanning microscopy (Carl Zeiss, Germany).

Cellular Fractionation

HCC cells were washed with PBS and scraped in 1 ml ice-cold PBS. Collect the cells and pellet cells at 130 *x g* in a refrigerated centrifuge at 4 °C for 3 min. Pellet cells were suspended in 5 volumes of ice-cold E1 buffer (50 mM HEPES-KOH pH 7.5, 140 mM NaCl, 1 mM EDTA pH8.0, 10% glycerol, 0.5% NP-40, 0.25% triton X-100, 1 mM DTT, 1x protease inhibitor cocktail). Then centrifuge and collect the supernatant as cytoplasm fraction, and the pellet was washed by E1 buffer for 2 times. After centrifugation at 1100 *x g* at 4 °C for 3 min, discarded the supernatant and resuspended the pellet by 2 volumes of ice-cold E2 buffer (10mM Tris-HCl pH8.0, 200mM NaCl, 1mM EDTA pH8.0, 0.5mM EGTA pH8.0, 1x protease inhibitor cocktail). Then centrifuge and collect the supernatant as nucleus fraction, and the pellet was washed by E2 buffer for 2 times. After centrifugation at 1100 *x g* at 4 °C for 3 min, discarded the supernatant and resuspended the pellet by E3 buffer (500mM Tris-HCl, 500mM NaCl, 1x protease inhibitor cocktail). The final solution (chromatin fraction) was sonicated for 5 min, and all fraction were centrifuged at 1600 *x g* at 4 ℃ for 10min and for western blot analysis.

Dual-luciferase reporter gene assay

The genomic promoter sequence, which was cloned into the pGL4.17 [luc/Neo] plasmid (Promega, USA) between -2000 and +100 base pairs (bp) from the cloned the

transcription start site (TSS) were synthesized by Wuhan Tsingke Company. The site-directed mutagenesis promoter sequence was also synthesized by Wuhan Tsingke Company. HCC cell lines were seeded into 24-well plates, then 480ng of pGL4.17 plasmid and 2ng of pRL-Renilla-luciferase plasmid were cotransfected into each well. After 48 h, cells were lysed in passive lysis buffer (Promega, USA) for 30 min and collected. The Dual-Luciferase Reporter Assay System (Promega, Madison, WI, USA) was used in accordance with the manufacturer's instructions to detect luciferase activity. Renilla activity was used to standardize the relative luciferase activity.

Chromatin immunoprecipitation (ChIP)

The SimpleChIP® Plus Sonication Chromatin IP Kit (Cell Signaling Technology #56383) was used for the chromatin immunoprecipitation (ChIP) assays. Briefly, HEK293T cells or HCC cell line were seeded in 15 cm dishes. The cells were then fixed, and the cell lysis was collected. The sonicator microprobe was used with the cell lysates for 4 rounds of 10 min, 1s pluses at output level 6. The shared chromatin was first incubated either with antibodies against CENPA or YY1 or with normal rabbit IgG overnight at 4°C, and followed by a 2h incubation with beads at the same temperature. The beads were washed with wash buffer four times, and then DNA was isolated for sequencing service or quantitative PCR. Sequencing service was provided by Bioyi Biotechnology Co., Ltd. Wuhan, China. The ChIP primers of CENPA and YY1 target genes are listed in Table S3.

Statistical Analysis

All experimental results were expressed as mean ± SEM. wherever applicable. Student's t-tests were used for comparisons between experimental and control conditions, and one-way ANOVA was used for multiple group comparisons. Statistical analyses were performed using SPSS (standard V.16.0; IBM Corporation, Armonk, NY). The Chi-square test was used to asses correlation between CENPA expression and the clinicopathological features of liver cancer patients. The correlation between CENPA, YY1, CCND1 and NRP2 expression were analyzed using Spearman's correlation test. The survival curves were compared with the Kaplan-Meier method and were compared with the log-rank test. The graphs' P values are shown by the asterisk symbols: $*P < 0.05$; $**P < 0.01$; $***P < 0.001$. A p value > 0.05 was considered not significant (n.s.) unless otherwise indicated.

Supplementary Figures

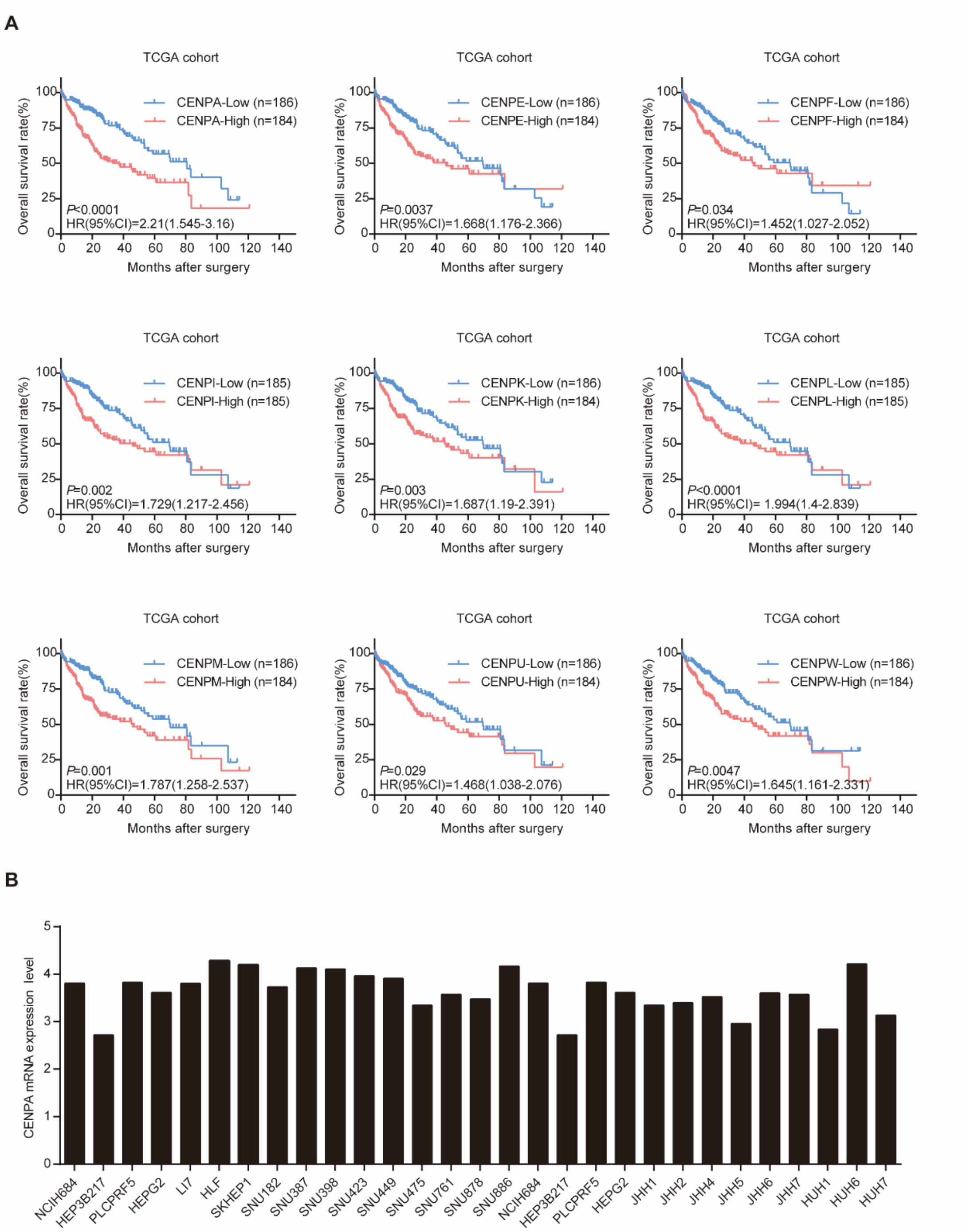

Figure S1. High expression of CENPA correlates with poor outcomes in patients with HCC.

(A) Kaplan-Meier curves of the OS rates among CENPs family members in TCGA database. **(B)** Relative CENPA expression levels in 29 HCC cell lines analyzed by the Cancer Cell Line Encyclopedia.

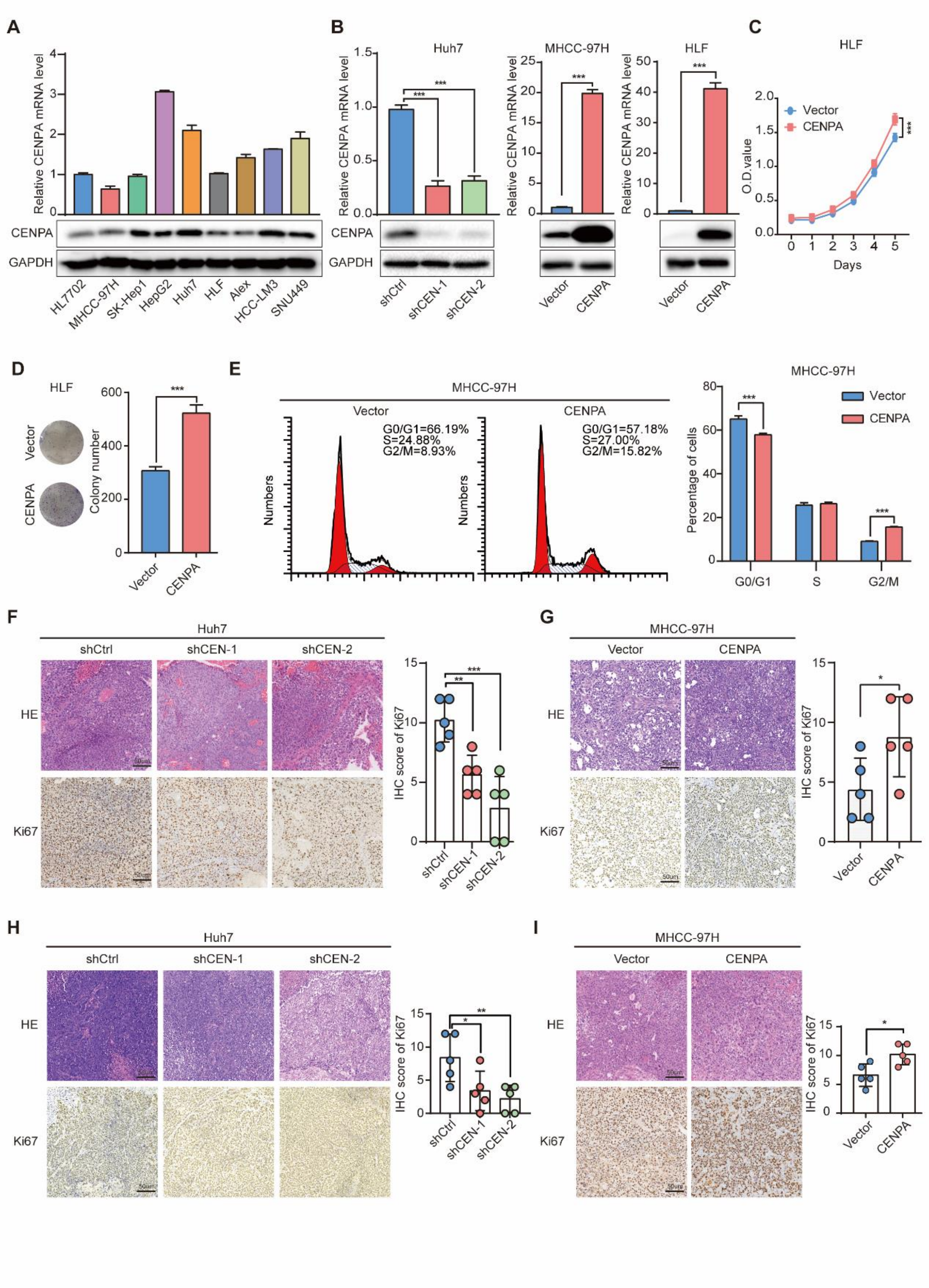

Figure S2. CENPA promotes HCC growth, proliferation, and cell cycle progression *in vitro* and *in vivo*.

(A) Relative CENPA mRNA and protein expression levels in HCC cells were analyzed

by real time PCR and western blotting. **(B)** The knockdown efficiency and overexpression of CENPA was verified by qPR-PCR and western blotting assays in Huh7, MHCC-97H and HLF cells. **(C)** CCK8 assay of HLF cells after CENPA overexpression cells. **(D)** Clone formation assays of HLF cells after CENPA overexpression cells. **(E)** Statistical analysis revealed the cell cycle changes after CENPA overexpression in MHCC-97H cells. **(F)** Representative images and quantification of HE staining and Ki67 staining of subcutaneous tumors from CENPA knockdown Huh7 cells (n=5 mice/group). **(G)** Representative images and quantification of HE staining and Ki67 staining of subcutaneous tumors from CENPA overexpression MHCC-97H cells (n=5 mice/group). **(H)** Representative images HE and Ki67 staining of orthotopic tumor from CENPA knockdown Huh7 cells (n=5 mice/group). **(I)** Representative images HE and Ki67 staining of orthotopic tumor from CENPA overexpression MHCC-97H cells (n=5 mice/group). The statistical significance was determined by Student's two-tailed t test. *$P < 0.05$; **$P < 0.01$; ***$P < 0.001$.

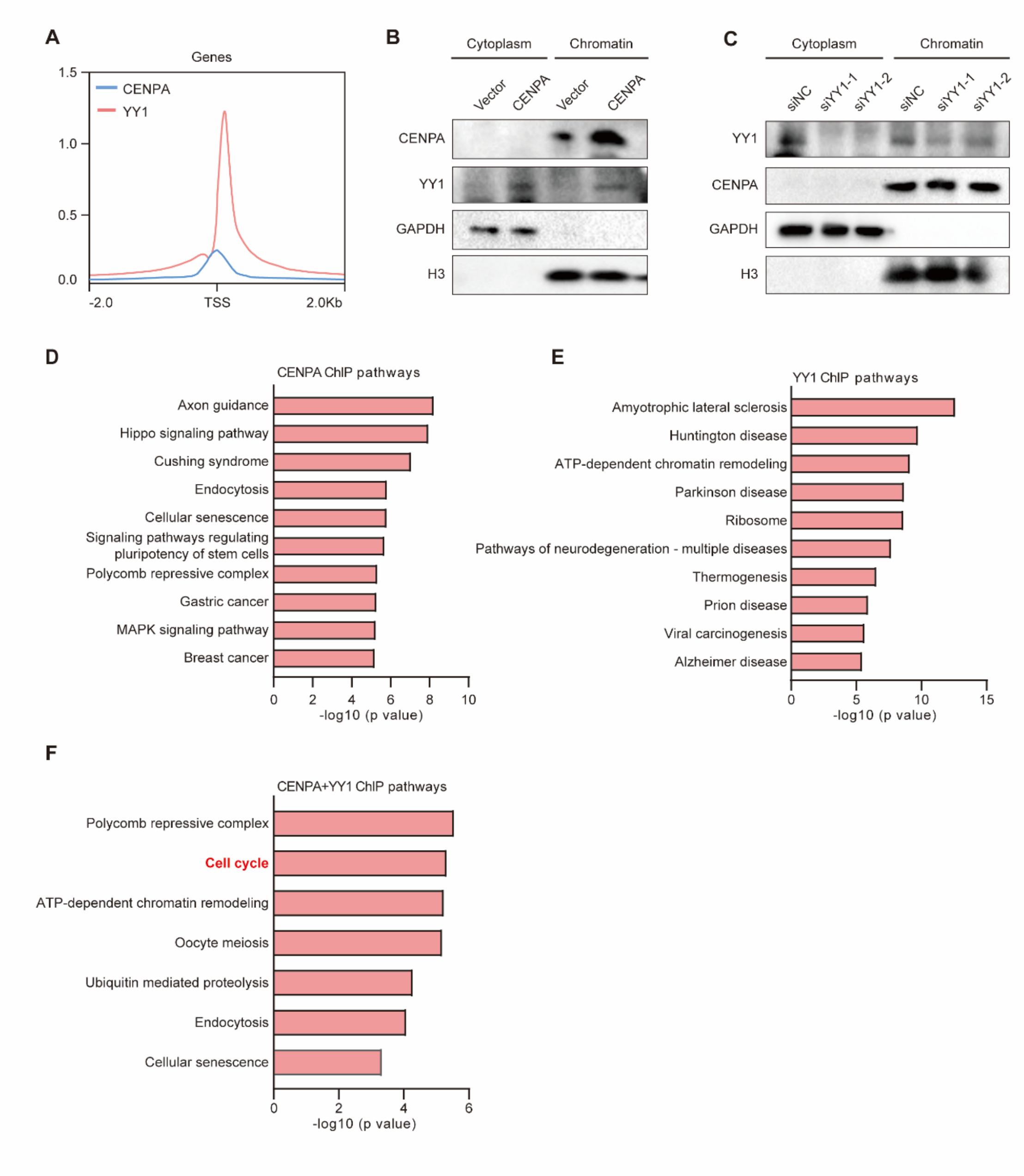

Figure S3. CENPA/YY1 collaborate as co-transcriptional factor.

(A) Peaks comparison of the overlapping regions suggested a co-binding model of CENPA and YY1. **(B)** Chromatin isolation of MHCC-97H after CENPA overexpression cells was performed to detect the abundance of CENPA and YY1 in cytoplasm and chromatin. **(C)** Chromatin isolation of MHCC-97H after YY1 knockdown cells was performed to detect the abundance of CENPA and YY1 in cytoplasm and chromatin. (GAPDH is the control for cytoplasmic fractionation; histone H3 is the control for

chromatin fractionation). **(D)** KEGG analysis showed signaling pathways regulated by CENPA. **(E)** KEGG analysis showed signaling pathway regulated by YY1. **(F)** KEGG analysis showed CENPA andYY1 co-regulate genes involving indicated pathways.

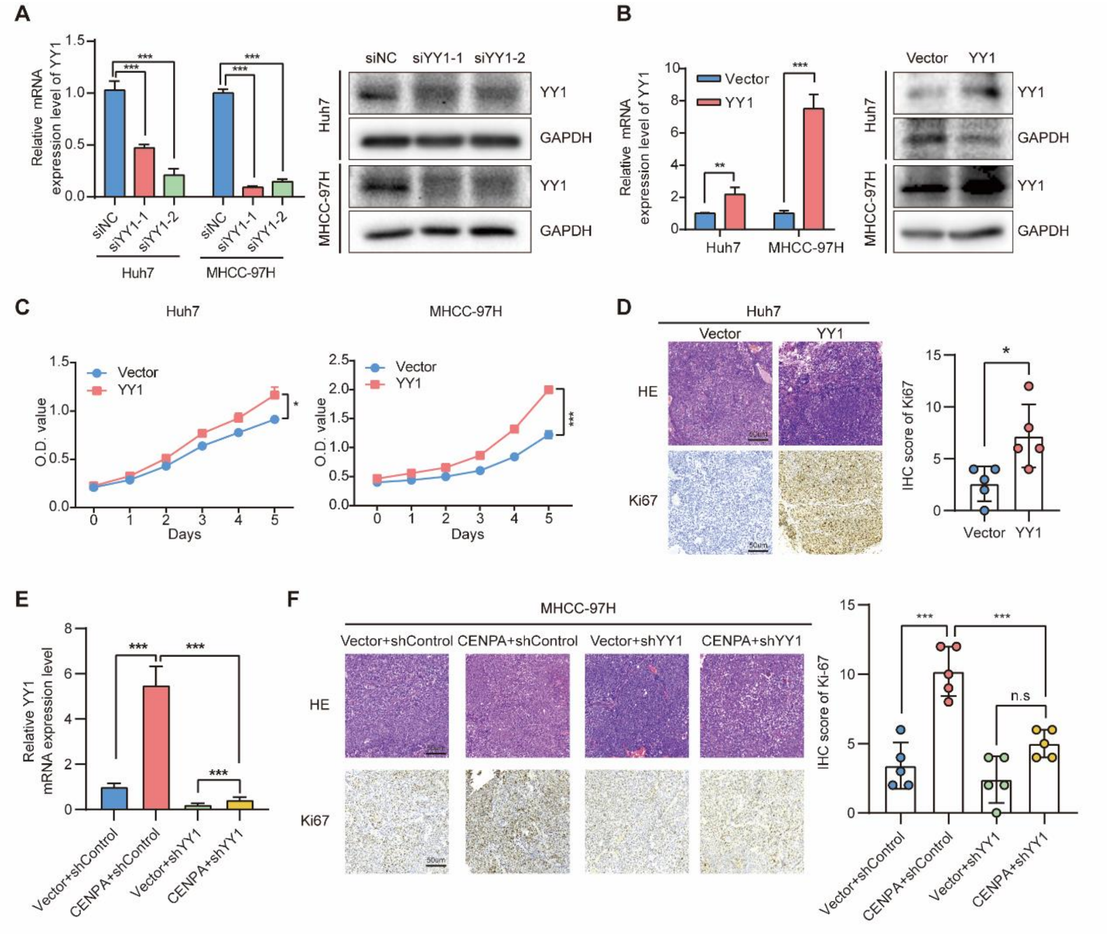

Figure S4. YY1 promotes HCC growth, proliferation, and cell cycle progression *in vitro* and *in vivo*.

(A) The knockdown efficiency of YY1 was verified by qRT-PCR and western blotting assays in Huh7 cells. **(B)** The overexpression efficiency of YY1 was verified by qRT-PCR and western blotting assays in MHCC-97H cells. **(C)** CCK8 assay in YY1 overexpression cells. **(D)** Representative images and quantification of HE staining and Ki67 staining of subcutaneous tumors of YY1 overexpression Huh7 cells. **(E)** The

knockdown efficiency of YY1 in CENPA overexpression MHCC-97H cells. **(F)** Representative images and quantification of HE staining and Ki67 staining of subcutaneous tumors. The statistical significance was determined by Student's two-tailed t test. *P < 0.05; **P < 0.01; ***P < 0.001.

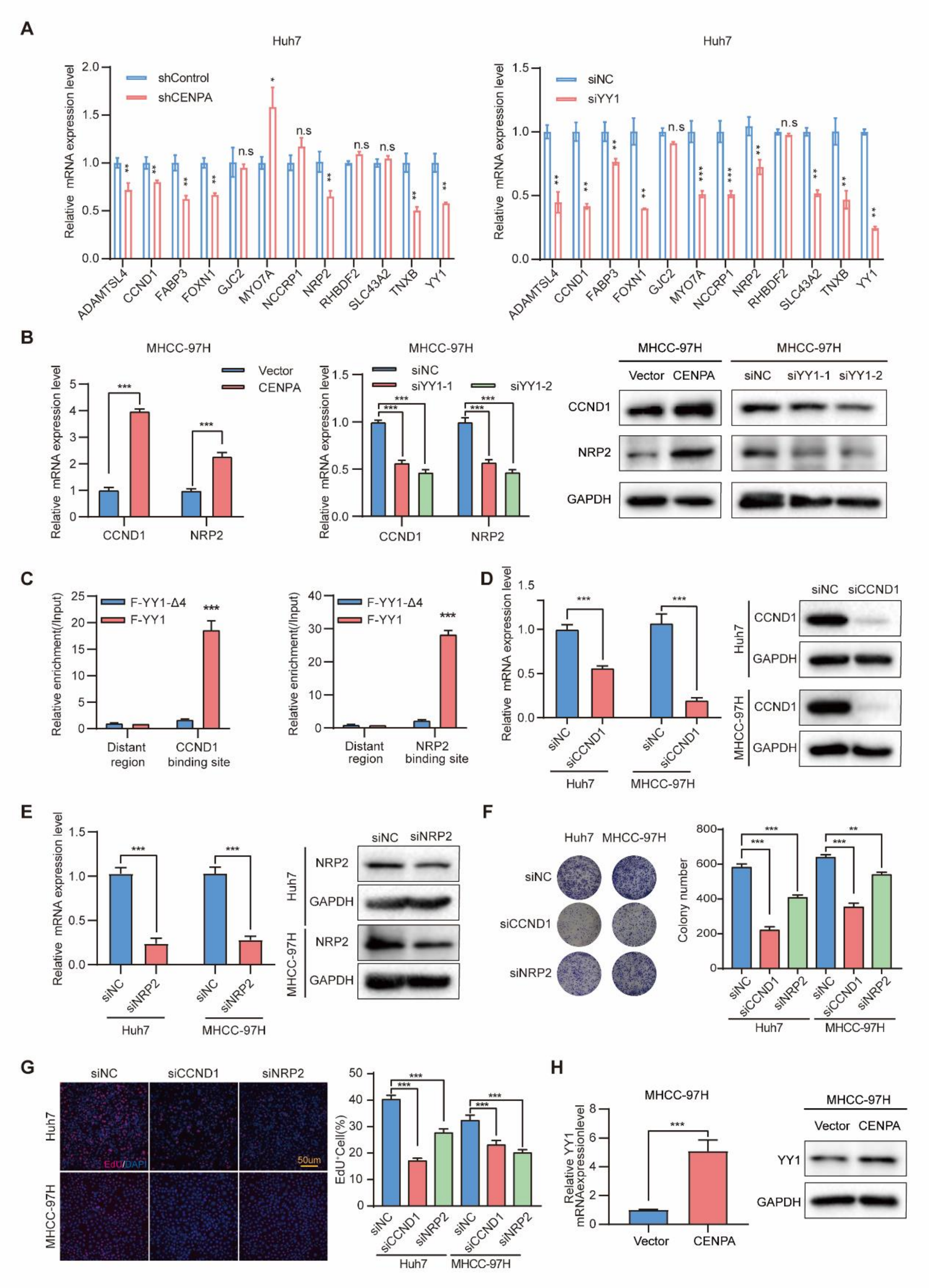

Figure S5. YY1 may serve as the target genes regulated by CENPA.

(A) Relative 12 target genes mRNA expression levels in Huh7 CENPA or YY1 knockdown cells were analyzed by real time PCR. **(B)** qRT-PCR and western blotting

detection of expression level of CENPA, YY1, CCND1 and NRP2 in MHCC-97H CENPA overexpression cells or YY1 knockdown cells respectively. **(C)** ChIP-qPCR assays demonstrated the binding of YY1 and YY1-Δ4 at the promoter regions of CCND1 and NRP2. **(D)** qRT-PCR and western blotting detection of expression level of CCND1 in Huh7 and MHCC-97H CCND1 knockdown cells respectively. **(E)** qRT-PCR and western blotting detection of expression level of NRP2 in Huh7 and MHCC-97H NRP2 knockdown cells respectively. **(F)** Clone formation assays of Huh7 and MHCC-97H cells after CCND1 or NRP2 knockdown cells. **(G)** Representative images of EdU assays and quantification of EdU^+ cells in Huh7 and MHCC-97H cells of CCND1 or NRP2 knockdown cells. **(H)** qRT-PCR and western blotting detection of expression level of YY1 in MHCC-97H CENPA overexpression cells. The statistical significance was determined by Student's two-tailed t test. *$P < 0.05$; **$P < 0.01$; ***$P < 0.001$.

Table S1. Gene name of CENPs family.

CENPs
CENPA
CENPB
CENPC
CENPE
CENPF
CENPH
CENPI
CENPJ
CENPK
CENPL
CENPM
CENPN
CENPO
CENPP
CENPQ
CENPR
CENPS
CENPT
CENPU
CENPV

CENPW

CENPX

Table S2. Candidate proteins identified by IP/MS assays.

Proteins
RPL7
RPS6
RPS8
RPL3;rpl3
RPL6
RPL18
RPL7A;RP-L7a
DKFZp781L0540;SF3B2
SEC16A
HEL-S-103;HSPA1B;HSPA1A
RPL35A
TUBB2C;TUBB4B;TUBB4A;TUBB8
YTHDF2
LTF;HEL110
DSG1
HEL-S-72p;HSPA8;HSPA2
PABPC1;PABPC3
SBSN
RPL13
HNRPUL1;HNRNPUL1

RPL4

TUBA1C;TUBA1A;TUBA1B;TUBA2;TUBA4A;TUBA3E;TUBA8;DKFZp686L0

YY1

GAPD;GAPDH;HEL-S-162eP

RPL30

RPLP0;RPLP0P6

JUP

RPL21

HIST1H1E;HIST1H1C;HIST1H1D

RPS18

IRS4

DDX3X;DDX3Y

EEF1A1;EEF1A1P5;EEF1A1L14;EEF1A2

RPL10;RPL10L

SERPINB12

RPL18A

RPL24;HEL-S-310

RPL8

RPL12;hCG_21173

PCMT1

RPL13A;RPL13a;RPL13AP3

RPL28

ANXA2;HEL-S-270;ANXA2P2

RPS27;RPS27L;LOC392748

RPL26;hCG_26523;RPL26L1

C7orf24;GGCT

SMARCC1

RPL27A;L27a

KHSRP

ENO1

RPS3

FLG

UBA52;UBB;RPS27A;UBC;HEL112;DKFZp434K0435;UbC

RPL27

KPRP

LYZ

TFG;TFG/ALK fusion

HNRPH3;HNRNPH3

LDHB;LDHC;LDHAL6A;HEL-S-133P;LDHA

RPL34

SMARCA4

TUBB;TUBB2B;TUBB2A;XTP3TPATP1;TUBB2C;DKFZp566F223;TUBB3

PKP1

RPS2;rps2;OK/KNS-cl.6;LOC392781;OK/KNS-cl.7

RPS14

PRDX1

RPL36

RPS23

RBBP4;RBBP7

RPL17;hCG_24487;RPL17-C18orf32

LARP1

RPS9

HEL-S-124m;HSPA9

RPS20

DKFZp451J085;YTHDF3;YTHDF1;DKFZp451A052

RPL23A

RPS25

ATP5B;HEL-S-271

H2AFV;HIST1H2AH;H2AFJ;HIST1H2AB;HIST1H2AK;HIST1H2AC;HIST1H2AJ;H2AFZ;HIST2H2AC;HIST2H2AB;HIST3H2A;HIST2H2AA3;HIST1H2AD;HIST1H2AG;HIST1H2AA;H2AFX

HNRNPH1;HNRNPH2

HEL-S-89n;HSPA5

HNRNPC;hCG_1641229

CRBN

TGM1

HEL-S-102;HSPB1

DNAJA2

HEL-S-49;TPI1

DSC1

HSP90AA1;EL52

LACRT

HEL-S-87p;ALDOA

CSTA

SMARCC2

RPSA;LOC388524

HNRNPM;HNRPM;ORF

PSMA8;PSMA7;hCG_41772

RTCB

S100A8

PRPF39

HJURP

RPS16

GTF2I;GTF2I-RARA

DDX17;DKFZp686J01190

MTA2;tmp_locus_6;MTA3

RPS26;RPS26P11

RPL37A;RPL37AP8

PUM1;PUM2

RPL35;LOC154880

H3F3B;H3F3A;HIST2H3PS2;H3F3C;HIST2H3A;HIST3H3;HIST1H3A

MOV10

RPL11

HEL-S-130P;CTSD

RPL32

HNRNPU;HNRPU

NCL

S100A9

RPL15

FGD6

RBM14

BLMH

S100A7;S100A7A

LTV1

RBM39;DKFZp686A11192;DKFZp781I1140;RNPC2;DKFZp781C0423;DKFZp6

86C17209

RPL19

FN3KRP

GPC1

MPO

SERPINA12

ZFYVE27

hCG_2024613;DNAJC9

S;CDSN

RPS19

CHD3

NOLC1

TXN

ZNF609

ZYG11B

LRRCC1

hCG_31253;FUBP3

DMKN

PPM1B

HEL-S-69p;PPIA

DDX1

HBA2;HBA1

PSMA3

GSG1

HESX1

SNC73;DKFZp686K04218;DKFZp686L19235;IGH@;DKFZp686M08189;DKFZp686C02218;DKFZp686O16217;DKFZp686C02220;DKFZp686G21220;DKFZp686J11235;DKFZp686K18196;IGHA2;IGHA1

HEL-S-2a;PRDX2

OK/SW-cl.65;DDX21

RPL37

SF1

RPS13

STOX2

GGH

SEC24C

TNRC6A

DAB1

CD38

RNF8

hCG_2014768;TMA7

CEP290

SEC13

VDAC2

RPL36A;RPL36A-HNRNPH2

NUFIP2

ZSCAN21;ZNF578;DKFZp686G16228;ZNF485;ZNF525;LOC125893;ZNF229;ZNF702P;ZNF320;ZNF468;ZNF765;ZNF701;ZNF599;ZNF813;ZNF860;ZNF816;ZNF28;ZNF888;ZNF761;ZKSCAN7;ZFP62;ZNF808;ZNF845;ZNF91

GSDMA

PRR4

hCG_37498;NPM1

RPL29

YWHAZ

CTSG

SPG20

SERPINB8

RAP1GAP;RAP1GA1

SYT5

TNRC6B

RPL14

FAM208B

RPS17

NDNL2

RPS4X

ETFDH

LOC100126582

PTPN4

TNNI2
ARHGAP25;KIAA0053;HEL-S-308
NEB
STK40
SPTB
OSBP
ABCA6
WUGSC:H_248O15.1;FRY

Table S3. **Primers, shRNAs, siRNAs, ChIP-qPCR primers and antibodies used in this study.**

Primers for qRT-PCR

**Gene**	**Sequence**
GAPDH Forward	5’-CTGGGCTACACTGAGCACC-3’
GAPDH Reverse	5’- AAGTGGTCGTTGAGGGCAATG -3’
CENPA Forward	5’-GACGCCTATCTCCTCACCTTA-3’
CENPA Reverse	5’-GTTGCACATCCTTTGGGAAGA-3’
YY1 Forward	5’-ACGGCTTCGAGGATCAGATTC-3’
YY1 Reverse	5’-TGACCAGCGTTTGTTCAATGT-3’
CCND1 Forward	5’-GCTGCGAAGTGGAAACCATC-3’
CCND1 Reverse	5’-CCTCCTTCTGCACACATTTGAA-3’
NRP2 Forward	5’-GCTGGCTATATCACCTCTCCC-3’
NRP2 Reverse	5’-TCTCGATTTCAAAGTGAGGGTTG-3’
ADAMTSL4 Forward	5’-CATCAAGCCAGGAATGTTCGG-3’
ADAMTSL4 Reverse	5’-AGGGGACGGAATAGCCTCTTC-3’
TNXB Forward	5’-GTGGTCCAGTATGAGGACACG-3’
TNXB Reverse	5’-CTGGTGGTCACGTCAGTCAC-3’
FABP3 Forward	5’-GGCACCTGGAAGCTAGTGG-3’
FABP3 Reverse	5’-CTGCCTGGTAGCAAAACCC-3’
FOXN1 Forward	5’-CTGCTCGTCATTTGTGTCCGA-3’
FOXN1 Reverse	5’-AGCCAAAGCCAGGATACTTGT-3’

GJC2 Forward	5'-GAGGTGCGACCGTTCTTTC-3'
GJC2 Reverse	5'-CTGACCACGTACATAACCAGC-3'
MYO7A Forward	5'-GGGACCATGTGTGGATGGAC-3'
MYO7A Reverse	5'-AGAGTCGCAGAGCTTCACCA-3'
NCCRP1 Forward	5'-ATTTCCGTGGCTGGTACATTAG-3'
NCCRP1 Reverse	5'-ATGGCTGGTTGTTCGTCATCC-3'
RHBDF2 Forward	5'-GATGGGGCAGACACGTTTGA-3'
RHBDF2 Reverse	5'-CCTCGGAAGTAGCTGGCAG-3'
SLC43A2 Forward	5'-AGTCAGAGGGCTTTTACTCCTAC-3'
SLC43A2 Reverse	5'-GTCCATGACGATACCCAGGG-3'

shRNA

**Gene**	**Sequence**
CENPA shRNA-1	5'-GCCTATCTCCTCACCTTACAT-3'
CENPA shRNA-2	5'-CCGAGTTACTCTCTTCCCAAA-3'
YY1 shRNA	5'-GGCAAGAAGAGUUACCUCATT-3'

siRNAs

**Gene**	**Sequence**
YY1 siRNA-1 Forward	5'-GGCAAGAAGAGUUACCUCATT-3'
YY1 siRNA-1 Reverse	5'-UGAGGUAACUCUUCUUGCCTT-3'
YY1 siRNA-2 Forward	5'-GAUGAUGCUCCAAGAACAATT-3'
YY1 siRNA-2 Reverse	5'-UUGUUCUUGGAGCAUCAUCTT-3'
CCND1 siRNA Forward	5'-CCACAGAUGUGAAGUUCAU-3'

CCND1 siRNA Reverse	5'-AUGAACUUCACAUCUGUGG-3'
NRP2 siRNA Forward	5'-CCAGAGCAUUUGUGCGCAA-3'
NRP2 siRNA Reverse	5'-UUGCGCACAAAUGCUCUGG-3'

ChIP-qPCR

**Gene**	**Sequence**
CCND1 BS Forward	5'-CAAGGACCGACTGGTCAAGG-3'
CCND1 BS Reverse	5'-TGTGCAAGTTTCATTCCGGC-3'
NRP2 BS Forward	5'-ATGAGACGCCTTCAAGCACC-3'
NRP2 BS Reverse	5'-AGGATTTCGTTACCCCTGCG-3'
YY1 BS Forward	5'-GCACCCCGCCGGGCGCTCGC-3'
YY1 BS Reverse	5'-GGCTCGCACGCGCCCTGGCT-3'

Antibodies

**Gene**	**Company**
CENPA	Abcam ab13939
	Cell Signaling Technology #2186
	Proteintech 26754-1-AP
CCND1	Abcam ab134175
YY1	Proteintech 22156-1-AP
NRP2	Abclonal A16061
Pan-KLa	PTM-1401RM
Anti-Flag Tag	Proteintech 20543-1-AP
Anti-HA Tag	Proteintech 51064-2-AP

GAPDH	Cell Signaling Technology #2118
H3	Proteintech 17168-1-AP
Ki67	Proteintech 27309-1-AP
